# Synthetic lipopeptides that interact with lipopolysaccharides are potent bactericidal compounds against *Xylella fastidiosa*

**DOI:** 10.1128/aem.00734-25

**Published:** 2025-07-30

**Authors:** Pau Caravaca-Fuentes, Laura Montesinos, Jordi Lamata, Marta Planas, Lidia Feliu, Emilio Montesinos

**Affiliations:** 1 Laboratory of Plant Pathology (CIDSAV), Institute of Agrifood Technology, University of Girona 16738, Girona, Spain; 2 LIPPSO, Chemistry Department, Institute of Agrifood Technology, University of Girona 16738, Girona, Spain; The University of Tennessee Knoxville, Knoxville, Tennessee, USA

**Keywords:** antimicrobial peptides, lipopeptides, LPS neutralization, plant pathogens, lytic effect

## Abstract

Novel strategies to control diseases caused by *Xylella fastidiosa* that affect important crops like grapevine, olive, almond, and citrus are necessary to prevent its establishment and spread in several countries. Target-oriented functional peptides toward the pathogen are potential new compounds, and peptides directed to lipopolysaccharides (LPS) are suitable candidates. In the present work, 36 peptides, including previously reported sequences and newly synthesized lipopeptides, were studied for their bactericidal and LPS neutralization activities, as well as for their hemolytic and phytotoxic activities. In a first stage, a simple and rapid method for detecting peptide-LPS interactions based on a chromogenic endotoxin assay was developed. A linear relationship was observed between LPS neutralization and the bactericidal activity. A strong LPS interaction was related to a high bactericidal activity, whereas peptides with slight or low interaction with LPS had low bactericidal activity. A selected group of lipopeptides that strongly interacted with the LPS was highly bactericidal with moderate-to-low hemolytic and phytotoxic activity (**BP389**, **BP473**, and **BP475**). These lipopeptides incorporate a butanoyl group at the side chain of a lysine residue and an L- or D-phenylalanine at position 4. The interaction and cell lytic effect between **BP473** and *X. fastidiosa* cells was confirmed by confocal microscopy experiments. These lipopeptides that target key components of the outer bacterial cell membrane are good candidates to mitigate infections by *X. fastidiosa* in plant hosts.

**IMPORTANCE:**

*Xylella fastidiosa* is a gram-negative bacterium that affects crops of economic importance such as grapevine, olive, almond, and citrus. The lack of efficient methods to control the diseases caused by this pathogen prompts the research of novel strategies. Since lipopolysaccharides (LPS) are a major virulence factor of *X. fastidiosa*, the development of new compounds that target LPS constitutes a promising approach. We identified cationic lipopeptides with a strong LPS interaction, high bactericidal activity, and low toxicity. These lipopeptides can be considered good candidates to control *X. fastidiosa* infections.

## INTRODUCTION

*Xylella fastidiosa* is a xylem-limited gram-negative plant pathogen that infects a wide range of plants. This bacterium causes significant economic losses by threatening the agricultural production of several crops worldwide and provoking high disease management costs and surveillance expenses (1–4). It infects several hundred plant species, including grapevine, olive, almond, and citrus (1, 5–7), and transmission occurs via insect vectors from Aphrophodidae or Cicadellidae families (8, 9). Following vector transmission, *X. fastidiosa* proliferates in the xylem, which induces its occlusion, causing a reduction of the hydraulic conductivity and resulting in severe symptoms, such as drought in the plant hosts (10, 11).

Currently, the main strategies to manage diseases caused by *X. fastidiosa* rely on quarantine and eradication measures as well as on vector control (12). Other methods are focused on the use of chemical compounds aimed to control the pathogen population in infected plants (13, 14) or to stimulate plant defense responses, but these strategies are not sufficiently effective (15). A promising approach to control plant pathogenic bacteria and fungi, including *X. fastidiosa,* is the use of natural peptides or synthetic analogs (16, 17). Within this field, the focus has nowadays shifted from the search for lytic peptides that destabilize bacterial membranes to a more refined strategy aimed at specific targets crucial for the pathogenicity of the bacteria (18).

In this context, our group has identified peptides not only with bactericidal activity against *X. fastidiosa* but also with antibiofilm activity or with the capacity to inhibit its motility or to stimulate the host plant defense responses (18–22). These sequences belong to several families of antimicrobial peptides developed in our group during the last two decades, with activity against other gram-negative plant pathogenic bacteria. These families include linear undecapeptides (CECMEL11 library) (23, 24), lipopeptides, peptide conjugates derived from the CECMEL11 library (25, 26), cyclic decapeptides (27), and KSL-W derivatives (28). An example is **BP178**, a multifunctional peptide with high bactericidal activity against *X. fastidiosa* and the capacity to stimulate plant defenses (18, 19). In addition, one of the best peptides against this pathogen is **1036**, which displays a dual activity (bactericidal and antibiofilm) (20). We have also found that **BP100** stands out for its capacity to inhibit twitching motility of *X. fastidiosa* (22). Moreover, **FV7** activates plant defense mechanisms of host plants of *X. fastidiosa* (21).

Targeting lipopolysaccharides (LPS) is a promising strategy to control plant pathogens. LPS, also called endotoxins, are part of the outer membrane of gram-negative bacteria and cover around 75% of the cell surface (29). They play a crucial role in the interaction between plant pathogens and their hosts and are essential for the virulence of plant pathogenic bacteria (30, 31). It has been described that in *X. fastidiosa*, the O-antigen from LPS delays the plant’s innate immune recognition, allowing the pathogen to evade the initial immune response (32). LPS also have an important contribution to cell-to-cell aggregation and play a crucial role in host colonization. Therefore, LPS constitute a major virulence factor in *X. fastidiosa* (29).

There have already been reported peptides with the capacity to interact with the LPS of bacteria. For instance, **YW12D**, **LBP-14**, **Lf(28–34)**, and **BPI(84–99)** share the capacity to neutralize LPS and, in mammals, are able to reduce inflammation caused by gram-negative bacteria (33–38). Peptides such as colistin and other polymyxins, a family of amphipathic peptides with a cyclic peptide core and a fatty acid tail, target LPS and facilitate cell lysis (39–42).

These findings prompted us to screen the antimicrobial peptides developed in our group for their capacity to neutralize LPS in *X. fastidiosa*. We selected peptides from the different families and evaluated their interaction with LPS as well as for their bactericidal activity against *X. fastidiosa* and their toxicity. A methodology to extract and quantify the LPS from *X. fastidiosa* was also developed together with an *in vitro* assay useful to identify peptides able to interact with LPS. In addition, the relationship between bactericidal activity and LPS neutralization was studied, and the best peptides were selected by their biological profile. Thus, the final goal of this work was to identify peptides able to neutralize LPS in *X. fastidiosa* as a suitable strategy to provide new lead compounds useful to control this pathogen.

## MATERIALS AND METHODS

### Synthesis of peptides

Peptides were synthesized manually on solid phase using polypropylene syringes of either 2 or 5 mL, which were equipped with a porous polyethylene disk. Removal of solvents and soluble reagents was accomplished through the application of suction. Solid-phase synthesis was performed following the standard 9-fluorenylmethoxycarbonyl (Fmoc)/*tert*-butyl (*t*Bu) strategy. A Fmoc-Rink-ChemMatrix resin (0.69 mmol/g) or a Fmoc-Rink-MBHA resin (0.56 mmol/g) was employed as a solid support. The Fmoc-Rink-ChemMatrix resin was chosen for the synthesis of peptides containing more than 11 residues. Peptide elongation was performed through sequential steps of Fmoc removal and coupling of the corresponding amino acid, as previously described (43). In the case of lipopeptides, the fatty acid chain was incorporated as previously described (26). Fluorescently labeled peptides were prepared incorporating 5(6)-carboxyfluorescein (CF) at the N-terminus (**BP473-CF**) or at the side chain of a lysine residue (**BP473-K[CF]**), which was coupled as Fmoc-Lys(Mtt)-OH. Upon completion of the peptide sequence, each resulting peptidyl resin was treated with trifluoroacetic acid (TFA)/H_2_O/triisopropylsilane (TIS) (95:2.5:2.5). Peptidyl resins that contained tryptophan and/or arginine were subjected to treatment with TFA/H_2_O/TIS/thioanisole/1,2-ethandithiol/phenol (81.5:5:1:5:2.5:5). Following TFA evaporation and diethyl ether extraction, the crude peptides were purified through reverse-phase column chromatography, lyophilized, analyzed by high performance liquid chromatography (HPLC), and characterized by mass spectrometry (Table S1).

#### Bacterial strains and growth conditions

Experiments were conducted within officially authorized laboratories that adhered to biosafety level II + under containment conditions as prescribed by the European and Mediterranean Plant Protection Organization (44) and the European Union (45). *X. fastidiosa* subsp. *fastidiosa* IVIA 5387.2 (ST1) isolated from almond in Mallorca (Spain) was used within the experiments (19).

The strain was stored in Pierce disease broth (PD2) (46) supplemented with glycerol at 30% and maintained at −80°C. When needed, the strain was cultured in buffered charcoal yeast extract (BCYE) agar plates (47) at 28°C for 7 days. Colonies were scrapped, and cells were cultured in fresh BCYE at 28°C for 7 additional days. In liquid cultures, PD3 was used (46). Cell suspensions were prepared in sterile succinate-citrate phosphate buffer for bactericidal experiments and in sterile phosphate-buffered saline (PBS) for LPS extractions. Suspensions were adjusted to 10^8^ cfu/mL (OD_600_ ~ 0.32) (20).

### Extraction of LPS from *X. fastidiosa*

Extraction of LPS from *X. fastidiosa* subsp. *fastidiosa* IVIA 5387.2 was performed using a lipopolysaccharide Isolation Kit (MAK339, Sigma-Aldrich, United States) following the manufacturer’s recommendations. Briefly, two tubes of 5 mL of an *X. fastidiosa* suspension with OD_600_ ≥ 0.6 were centrifuged two times. Then, lysis buffer was added; lysate was sonicated with a Sonifier S450D (Branson, United States), incubated on ice, and centrifuged. Proteinase K was added, and the tube was heated at 60°C. Finally, the lysate was centrifuged, and the lysate was transferred to a fresh tube and stored at −20°C.

Purity of LPS was evaluated on a Criterion Precast gel 16.5% Tris-Tricine/Peptide 1.0 mm (Bio-Rad, United States).

Coomassie blue G-stained LPS bands were viewed using the Image Lab Software (6.1) included in the Chemidoc XRS + System (Bio-Rad, USA).

### LPS-peptide neutralization

LPS-peptide neutralization assays were set up and performed using the Pierce Chromogenic Endotoxin Quant Kit (Thermo Fisher Scientific, United States) in 96-well plates following the manufacturer’s recommendations. Briefly, standard curves were prepared and validated with a lyophilized *Escherichia coli* (O111:B4) endotoxin standard (from 0.1 to 1 EU/mL), and the optical density at 405 nm (OD_405_) was measured using Varioskan Flash (Thermo Fisher Scientific, United States). The degree of interaction was measured by the ratio (*R*) between OD_405_ of treatments (OD_T_) and OD_405_ of the non-treated control (OD_NTC_). *R* was calculated following the equation: *R* = ([OD_T_ − OD_B_]/[OD_CNT_ − O_B_]), where OD_B_ is OD_405_ of endotoxin-free water. Each sample was conducted in triplicate.

To identify the best reference peptide to be used in the assays, peptides with previously described LPS interaction (Table 1) were mixed at a final concentration of 25 µM with *E. coli* LPS at 1 EU/mL or 0.5 EU/mL, diluted in endotoxin-free water, and tested as described previously.

**TABLE 1:** Sequences and references of the selected peptides

Code	Sequence^*a*^	Reference
Linear undecapeptides (CECMEL11)		
BP013	FKLFKKILKVL-NH_2_	48
BP015	KKLFKKILKVL-NH_2_	48
BP016	KKLFKKILKKL-NH_2_	48
BP022	Ac-LKLFKKILKVL-NH_2_	48
BP076	KKLFKKILKFL-NH_2_	48
BP100	KKLFKKILKYL-NH_2_	23
Linear undecapeptides derived from BP100 containing a D-amino acid		
BP143	KKLfKKILKYL-NH_2_	24
BP144	KKlFKKILKYL-NH_2_	24
BP145	KkLFKKILKYL-NH_2_	24
BP146	KKLFKkILKYL-NH_2_	24
Lipopeptides derived from BP100		
BP375	Ac-KKLFKKIK(COC_5_H_11_)KYL-NH_2_	25
BP377	Ac-KKLFKKILKK(COC_5_H_11_)L-NH_2_	25
BP387	Ac‐KKLFKKIK(COC_3_H_7_)KYL‐NH_2_	25
BP389	Ac-KKLFKKILKK(COC_3_H_7_)L-NH_2_	25
BP393	Ac-KK(COC_11_H_23_)LFKKILKYL-NH_2_	25
BP473	Ac-KKLfKK(COC_3_H_7_)ILKYL-NH_2_	26
BP474	Ac-KKLfKKIK(COC_3_H_7_)KYL-NH_2_	26
BP475	Ac-KKLfKKILKK(COC_3_H_7_)L-NH_2_	26
BP485	C_3_H_7_CO-KKLfKKILKYL-NH_2_	26
BP490	Ac-KKLfKKIK(COC_11_H_23_)KYL-NH_2_	26
BP494	Ac-KKLfKKK(COC_5_H_11_)LKYL-NH_2_	26
BP495	Ac-KKLfKKILKYK(COC_5_H_11_)-NH_2_	26
BP496	Ac-KKLfKKILKYK(COC_3_H_7_)-NH_2_	26
BP498	Ac-KKLfK(COC_3_H_7_)KILKYL-NH_2_	26
BP499	Ac-KKLfKKILK(COC_3_H_7_)YL-NH_2_	26
BP500	Ac-KKK(COC_11_H_23_)fKKILKYL-NH_2_	26
BP501	Ac-KKLHKKILKK(COC_3_H_7_)L-NH_2_	This work
BP545	Ac-K(COC_3_H_7_)KLfKKILKYL-NH_2_	This work
BP546	Ac-KKK(COC_3_H_7_)fKKKLKYL-NH_2_	This work
BP547	Ac-KKLk(COC_3_H_7_)KKILIYL-NH_2_	This work
BP548	Ac-KKLfKKK(COC_3_H_7_)LKYL-NH_2_	This work
BP549	Ac-KKK(COC_3_H_7_)fKKILKYL-NH_2_	This work
BP550	Ac-KKLk(COC_3_H_7_)KKILKYL-NH_2_	This work
Cyclic decapeptide (CYCLO10)		
BPC098W	c(LLKKKWKKLQ)	27
Peptide analog of KSL-W		
BP442	KKVVFWVKFk-NH_2_	28
Peptide conjugate derived from BP100		
BP178	KKLFKKILKYLAGPAGIGKFLHSAKKDEL-OH	49
Peptides described with LPS-neutralizing activity		
BPI(84-99)	NIKISGKWKAQKRFLK-NH_2_	33, 34
LBP-14	RVQGRWKVRASFFK-NH_2_	35, 36
Lf (28–34)	RKVRGPP-NH_2_	37, 38
YW12D	YVKLWRMIKFIR-NH_2_	38
CF-labeled BP473 analogs		
BP473-CF	CF-KKLfKK(COC_3_H_7_)ILKYL-NH_2_	This work
BP473-K(CF)	Ac-K(CF)KKLfKK(COC_3_H_7_)ILKYL-NH_2_	This work

^*a*^ Lowercase letters indicate a D-amino acid; Ac = acetyl.

To adjust peptide concentration for the LPS-peptide assays, **YW12D** peptide (used as reference) at different final concentrations (50, 100, 150, and 200 µM) was mixed with *E. coli* LPS at 0.5 EU/mL. Upon selection of the desired concentration, selected peptides were mixed at a final concentration of 150 µM with *E. coli* LPS at 0.5 EU/mL and tested. To ensure the peptides do not shield the OD_405_ signal through some polar or similar residues, most of them were also evaluated without LPS.

For comparison purposes with *E. coli* LPS, some peptides were tested at a final concentration of 150 µM using LPS extracted from *X. fastidiosa* subsp. *fastidiosa* IVIA 5387.2. In this case, the LPS concentration was adjusted following kit recommendations by preparing serial dilutions and comparing to the standard curve made with *E. coli* (O111:B4) endotoxin. Each sample was conducted in triplicate.

### Bactericidal activity

The bactericidal activity of peptides was assessed by a contact test coupled with viable PCR (v-qPCR) as previously described (50). The v-qPCR permits the quantification of viable cells, discarding dead or non-viable cells, thanks to PMA, a nucleic acid binding dye. Sensitivity and amplification efficiency of the v-qPCR and standard curves were previously evaluated and set up for the studied strain (20).

Peptides were solubilized in sterile Milli-Q water to a stock concentration of 1 mM and filter sterilized through a 0.22 µM pore size filter. Briefly, the bactericidal activity of the selected peptides was determined by a 3 h contact test at room temperature with a 10^7^ cfu/mL *X*. *fastidiosa* suspension. PMA was added, and samples were incubated and processed following previously described protocols (50). The reduction of viability was obtained as described previously (20). Peptides were tested generally at a final concentration of 50 µM. Highly active peptides (those causing a reduction in viability ≥2 logs) were further tested at 12.5 and 3.1 µM to better characterize their bactericidal activity. **BP377**, **BP393**, and **BP473** were tested at 3.1, 6.25, 12.5, 25, and 50 µM to study their dose-response effect. All treatments were performed in triplicate.

### Phytotoxicity

Peptides were evaluated for their phytotoxicity as previously described (49). Tobacco plants (*Nicotiana tabacum*) were grown from seed in a glasshouse between 20 and 30 days. A total of 100 µL of each peptide at 150 µM was infiltrated into the mesophylls of fully expanded tobacco leaves. For each peptide and concentration, at least three leaves randomly distributed along distinct plants were similarly infiltrated. For the purposes of control, infiltrations with water (negative control) or with the highly phytotoxic melittin peptide (positive control) were performed at the same concentration. After 48 h, the phytotoxicity was quantified as the lesion diameter.

### Hemolytic activity

The hemolytic activity of peptides was evaluated by determining hemoglobin release from erythrocyte suspensions of horse blood (5% [vol/vol]; Thermo Fisher Scientific, Spain) as previously described (23). In brief, the peptides were dissolved in a TRIS buffer and subsequently mixed with purified horse erythrocytes that were diluted 10-fold. The peptides were tested at final concentrations of 50 and 250 µM. The hemolysis percentage (*H*) was determined using the mathematical equation: *H* = 100 × ([Op − Ob]/[Om − Ob]), where Op is the density for a given compound concentration, Ob is for the buffer, and Om is for the melittin-positive control.

### Confocal microscopy

Cell suspensions of *X. fastidiosa* at 10^7^ cfu/mL were treated with CF and CF-labeled peptides and incubated at different times (30 s, 5 min, 10 min, and 30 min). Then, adapted from previously described protocols (51–53), paraformaldehyde was added for fixation, reaching a final concentration of 2%, incubated at room temperature for 20 min followed by two washes with PBS and stored at 4°C until cell observation.

Confocal microscopy was performed with a NIKON Ti Eclipse motorized inverted microscope with a NIKON A1R confocal module (NIKON, Japan; Research Technical Services from the University of Girona). Images were processed and compared with the software NIS elements v4.10 (NIKON, Japan).

### Data analysis

Statistical significance in the assays comparing techniques for detecting peptide-LPS interactions was assessed using a two-way analysis of variance (ANOVA), considering treatments (peptides) and techniques (LPS from *X. fastidiosa* or *E. coli*) as the two factors. The significance of the effect of peptides in LPS neutralization and their bactericidal activity against *X. fastidiosa* was evaluated by one-way ANOVA. Means were separated according to Duncan’s test at a *p*-value of <0.05 (IBM Statistics for Windows, Version 29.0 released in 2023 by IBM Corp, Armonk NY, United States).

The relationship between the LPS neutralization capacity and bactericidal activity was analyzed using a simple linear regression model and was fitted with MATLAB (MathWorks, Natick, MA). The software was used to calculate the regression line parameters, the coefficient of determination (*R*^2^), and the *p*-value for the test of whether the slope differs significantly from zero.

In lipopeptides, for every variable studied (bactericidal activity, LPS neutralization, hemolysis, and effect on tobacco plants), a threshold was arbitrarily determined to separate peptides in different biological activity profiles: 2.0 LogN_0_/N for bactericidal activity, 0.25 ratio for LPS neutralization, 10% for hemolytic activity, and 7 mm for the effect on tobacco plants.

For dose-response assays, the half-maximal effective concentration (EC_50_) of the peptides was calculated using EC_50_ calculator with the two-parameter model (ATT Bioquest, Pleasanton, USA).

## RESULTS

### Selection and synthesis of peptides

The 36 peptides selected for this study are shown in Table 1. They belong to the following peptide families: (i) linear undecapeptides from CECMEL11 library; (ii) linear undecapeptides derived from **BP100** containing a D-amino acid; (iii) lipopeptides derived from **BP100**, including seven sequences not previously described; (iv) a cyclic decapeptide from the CYCLO10 library; (v) a peptide analog of **KSL-W;** (vi) a peptide conjugate derived from **BP100**. In addition, four previously reported peptides from other sources, with the capacity to neutralize LPS, were also selected as positive controls. Furthermore, lipopeptide **BP473** was fluorescently labeled at the N-terminus (**BP473-CF**) or at the side chain of an additional Lys incorporated at the N-terminus (**BP473-K[CF]**). All peptides were manually synthesized and purified, being obtained in HPLC purities >90% except for **BP015**, **BP143**, **BP144**, and **BP146** (purities >80%). Peptide identity was confirmed by high-resolution mass spectrometry (Table S1).

### Activity of peptides on LPS neutralization

The Pierce Chromogenic Endotoxin Quant Kit was first tested to ensure the detection of free LPS and the achievement of a reduction in the ratio of free LPS in the presence of LPS-neutralizing peptides. To set up the LPS neutralization assay, a standard curve was validated following the manufacturer’s recommendations, obtaining the equation OD_405_ = 1.039*X* – 0.034, where *X* is LPS concentration expressed as EU/mL, and with *R*^2^ = 0.9924.

Peptides with previously described LPS neutralization activity (**YW12D**, **LBP-14**, **Lf[28–34)**, and **BPI[84–99]**) were tested with *E. coli* 0111:B4 LPS at 0.5 and 1 EU/mL (Fig. S1). A lower OD_405_ ratio between peptide-treated samples and NTC was observed at 0.5 EU/mL, and this concentration was selected for the next assays. The peptide that showed the lowest OD_405_ ratio was **YW12D**, which was chosen for the next experiment to determine in more detail the optimal peptide concentration to perform the LPS neutralization assays. Thus, **YW12D** was evaluated at concentrations ranging from 25 to 200 µM (Fig. S2). It was considered that the OD_405_ ratio obtained at 150 µM was adequate to carry out the LPS neutralization assays for screening the 36 selected peptides (Table 1).

**Fig 1:**
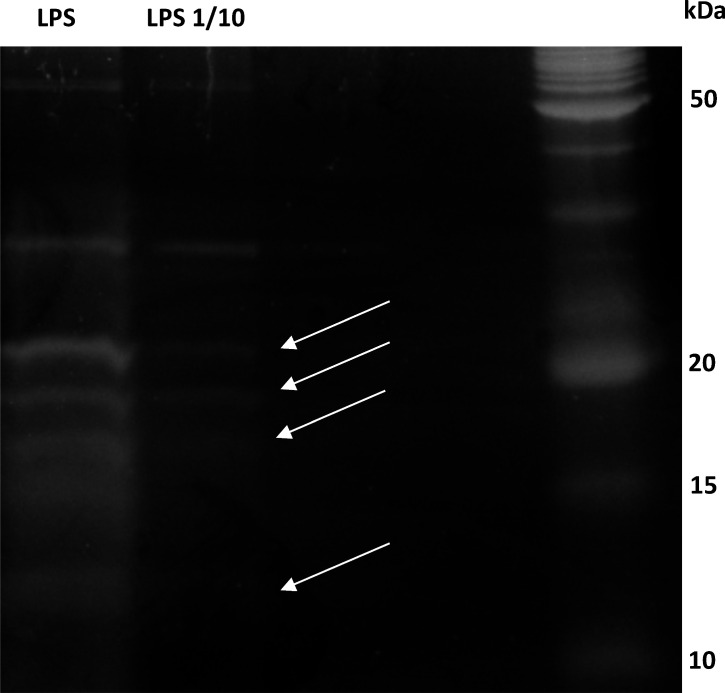
Electrophoresis gel showing extracts of LPS from *X. fastidiosa subsp. fastidiosa*. Direct LPS extract (lane 1), a 1/10 dilution (lane 2), and the molecular marker (lane 3). White arrows correspond to expected bands with LPS weights according to the extraction kit.

Next, LPS from *X. fastidiosa* subsp. *fastidiosa* IVIA 5387.2 were extracted and partially purified by electrophoresis (Fig. 1). Bands corresponding to LPS were identified. The resulting extracts were highly concentrated, and their dilution was required. These extracts were quantified using the standard curve obtained from the *E. coli* assays. An equivalent LPS concentration of 0.4 EU/mL for a 10^−7^ dilution of the extracts was found as the most appropriate for the assays.

**Fig 2:**
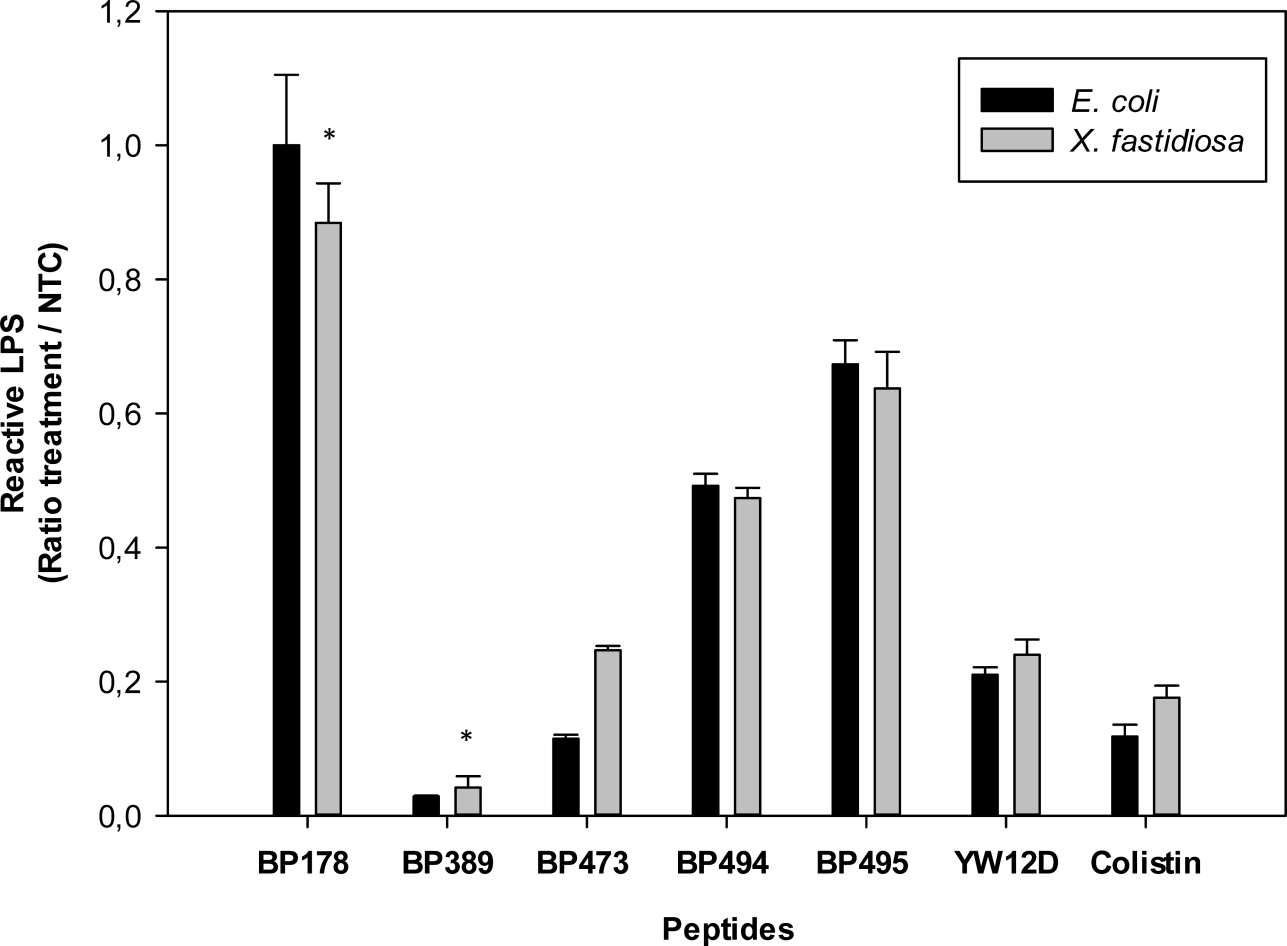
Effect of selected peptides at 150 µM on LPS neutralization. Bars correspond to assays using lyophilized LPS from *E. coli* 0111:B4 (black bars) or *X. fastidiosa subsp. fastidiosa* IVIA 5387.2 (gray bars) both at 0.5 EU/mL. Values are the means of three replicates, and error bars represent the confidence interval α = 0.05. Significant differences between techniques within each treatment according to a two-way ANOVA are indicated by asterisks (*P* < 0.05).

Once the conditions of the test were set up, the LPS neutralization capacity of seven peptides (colistin, **YW12D**, **BP178**, **BP389**, **BP473**, **BP494**, and **BP495**) was analyzed with LPS from both *E. coli* 0111:B4 and *X. fastidiosa* subsp. *fastidiosa* IVIA 5387.2 (Fig. 2). Colistin and **YW12D** were used as LPS-neutralization reference peptides.

In the two-way ANOVA assessing the effect of treatment and bacterial LPS (*X. fastidiosa* or *E. coli*) on LPS neutralization, the main effect of treatment was highly significant (*F* = 1,020.253, *P* < 0.001), confirming that different peptides have clearly distinct LPS-binding capacities. The main effect of bacterium LPS was not significant (*F* = 0.000, *P* = 0.990), suggesting that, overall, the two bacterial LPS systems responded similarly to the treatments. However, the interaction effect between treatment and bacterial LPS was statistically significant (*F* = 15.382, *P* < 0.001), indicating that in some cases, the response to a given peptide depended on the bacterial LPS system used.

To explore this further, we performed univariate analyses of simple effects (per treatment) to compare responses between the two bacterial LPS models. These analyses showed that only two treatments (**BP473** and **BP178**) presented statistically significant differences between bacterial LPS systems (*p* < 0.001). For the remaining treatments, no significant differences were observed (*p* > 0.05). This indicates that, in most cases, the two models produce comparable LPS neutralization profiles. Despite the differences observed in the interaction effect, both techniques (*X. fastidiosa* and *E. coli*) are able to discriminate peptides with very high, high, moderate, and low LPS-binding capacity. Therefore, it was concluded that peptide-LPS neutralization assays performed with LPS extracted from *X. fastidiosa* were equivalent to those carried out using the commercial kit containing *E. coli* LPS. Thus, the next experiments were run with this kit.

**Fig 3:**
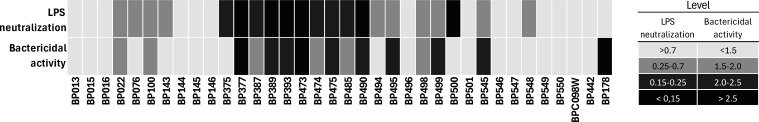
LPS neutralization and bactericidal activity of the 36 peptides screened.

The LPS interaction of the 36 antimicrobial peptides and the four reference peptides with previously described LPS interaction was analyzed with the above kit (Table S2). Peptides were classified into four different groups (Fig. 3). The first group included six lipopeptides (**BP377**, **BP389**, **BP393**, **BP473**, **BP490**, and **BP500**) with a very high LPS neutralization level (a ratio <0.15). The second group comprised five lipopeptides (**BP375**, **BP387**, **BP474**, **BP475**, and **BP485**) with a high activity (a ratio between 0.15 and 0.25). The third group included 10 peptides with a moderate activity (a ratio between 0.25 and 0.7), and the rest of the peptides showed low LPS neutralization (a ratio higher than 0.7). Peptides previously described with LPS neutralization activity exhibited moderate to high levels of LPS interaction (a ratio between 0.21 and 0.30).

### Bactericidal activity of peptides

Bactericidal activity of the 36 peptides was determined at a concentration of 50 µM. The peptides were classified into four statistically different groups (Fig. 3; Table S2). Peptide **BP178** and lipopeptides **BP377** and **BP473** showed very high bactericidal activity (>2.5 log), and lipopeptides **BP389**, **BP393**, **BP475**, **BP490**, **BP495**, **BP499**, and **BP545** displayed high bactericidal activity (between 2.0 and 2.5 log). Six peptides exhibited a moderate activity (between 1.5 and 2.0 log), and the rest of the sequences were low active (<1.5 log). The most active peptides were assayed at 12.5 and 3.1 µM, and notably, **BP178**, **BP473**, **BP490**, and **BP499** showed high activity at 12.5 µM.

The bactericidal activity of the CF-labeled **BP473** analogs (**BP473-CF** and **BP473-K[CF]**) was studied at 25 µM (Table S2). **BP473-K(CF)** was the most active analog (1.78 ± 0.053 log) and was selected to perform the confocal microscopy assays.

**Fig 4:**
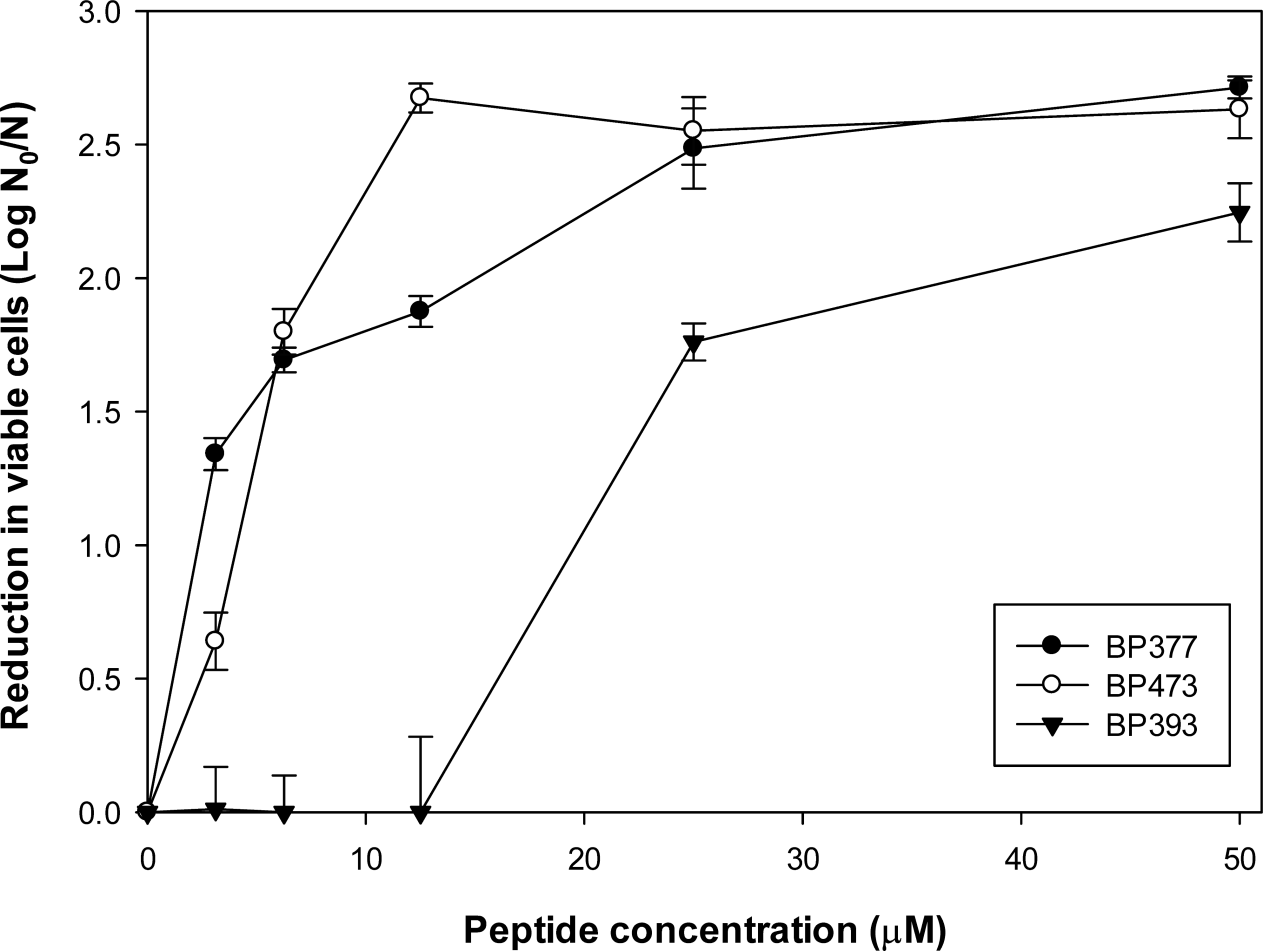
Dose-response relationship between the reduction in viability of *X. fastidiosa subsp. fastidiosa* IVIA 5387.2 and peptide concentration after 3-hour contact with selected peptides. N_0_ is 10^7^ cfu. Values are the means of three replicates, and error bars represent confidence interval (α = 0.05).

Three selected peptides with a high LPS-neutralizing level (**BP377**, **BP393**, and **BP473**) were studied for bactericidal activity in more detail at concentrations ranging from 3.1 to 50 µM, indicating a saturation relationship (Fig. 4). The median effective concentration (EC_50_) for the peptides was low for **BP377** and **BP473** (3.67 µM ± 0.23 and 4.75 µM ± 0.06, respectively), indicating high activity, compared to **BP393** (20.11 µM ± 1.16).

### Relationship between bactericidal activity and LPS neutralization

**Fig 5:**
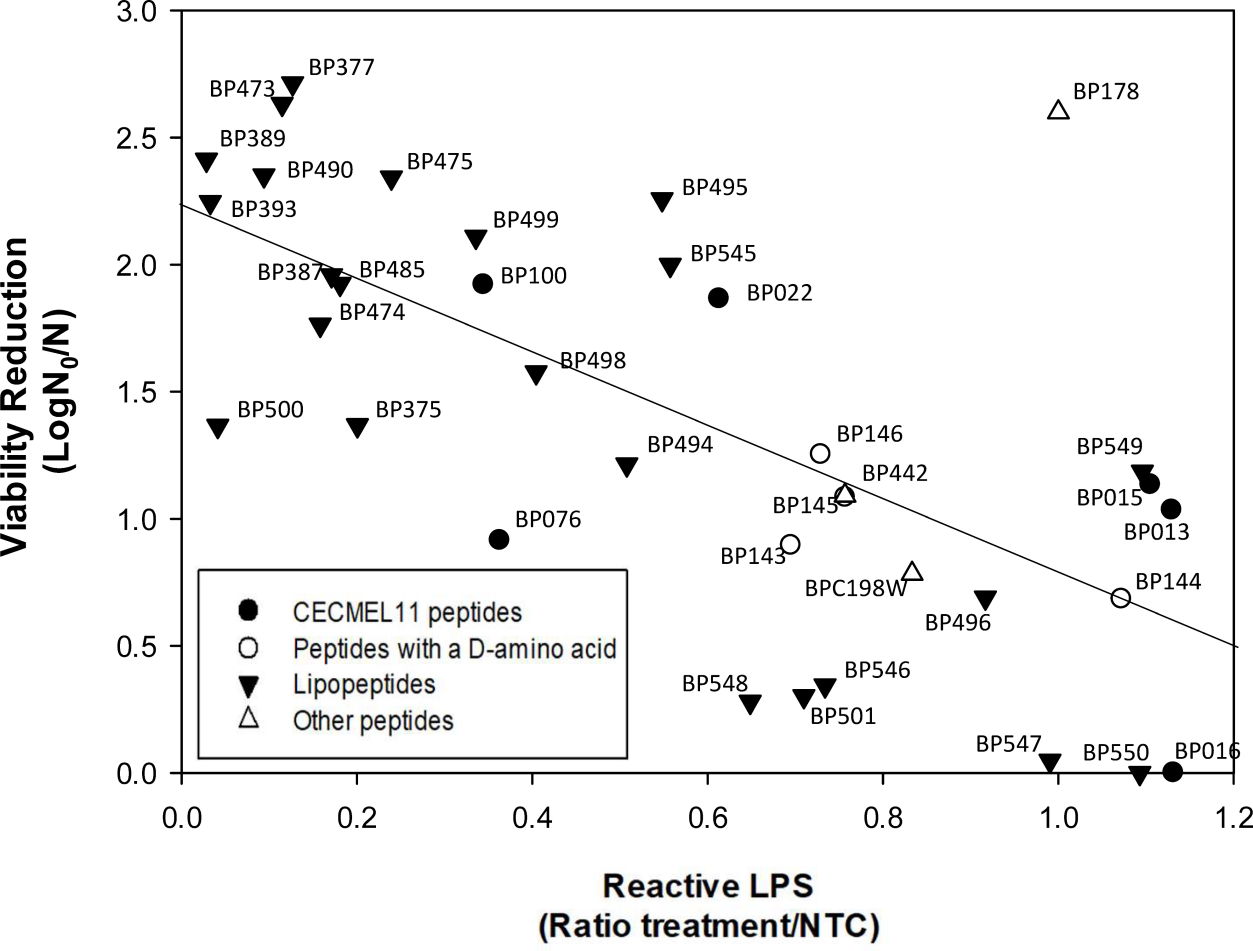
Relationship between bactericidal activity of peptides against *X. fastidiosa* and LPS neutralization. The regression equation is *y* = −1.63X + 2.26; *R*^2^ = 0.582 and *P*-value = 1.57 × 10^−7^. **BP178** has been excluded from the regression as an outlier.

Among the 36 peptides studied, a moderate linear relationship (*R*^2^ = 0.582, *P* < 0.01) was observed between the bactericidal activity and the capacity to neutralize LPS (Fig. 5), with lipopeptides showing high LPS neutralization capacity generally exhibiting higher bactericidal activity. Particularly, the best sequences with both activities were lipopeptides **BP377**, **BP389**, **BP393**, **BP473**, and **BP490**, which displayed viability reduction levels >2 log and a ratio of reactive LPS <0.2.

### Biological profile of lipopeptides

**Fig 6:**
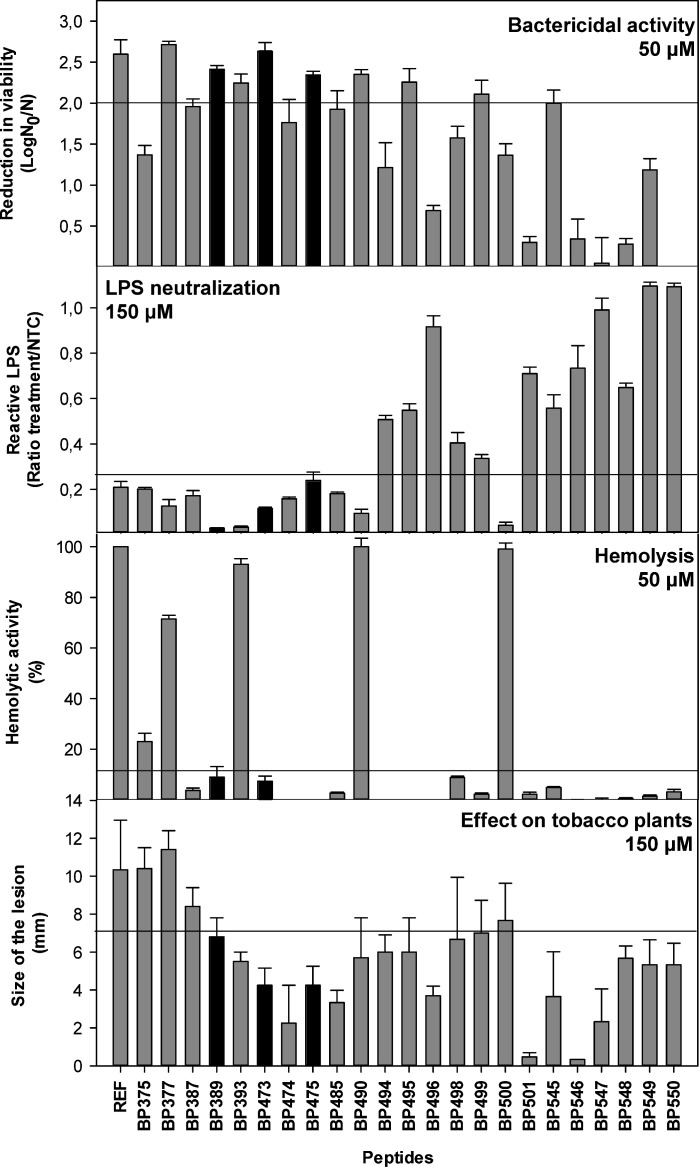
Biological activity of lipopeptides. Bactericidal activity against *X. fastidiosa*, LPS neutralization, hemolysis, and effect on tobacco leaves. Values are the means of three replicates, and error bars represent the confidence interval (α = 0.05). REF indicates reference compounds: **BP178** at 25 µM for bactericidal activity, **YW12D** for LPS neutralization, and melittin for hemolysis and effect on tobacco leaves. Horizontal lines represent arbitrarily defined thresholds used to select peptides based on their biological activity profiles. Black bars highlight peptides with high bactericidal activity, high LPS neutralization, and low toxicity.

The biological activity profile of the 23 lipopeptides was further studied because most of them showed a good relationship between bactericidal activity and LPS neutralization. They were screened for their hemolytic activity and phytotoxicity (Fig. 6). Eighteen lipopeptides showed a hemolytic activity ≤10% at 50 µM (Table S3). Nineteen lipopeptides showed lesions ≤7 mm at 150 µM when infiltrated into the mesophylls of tobacco leaves (Table S3).

The biological profile of the 23 lipopeptides was analyzed by comparing their bactericidal activity, LPS neutralization, hemolytic activity, and leaf infiltration effect (Fig. 6). To classify these lipopeptides in groups, the following arbitrary threshold values for each of these activities were established: a reduction of viability ≥2 log (high bactericidal activity), a ratio of reactive LPS ≤0.25 (high LPS neutralization), ≤10% hemolysis (low hemolytic activity), and diameter of leave lesion ≤7 mm (low phytotoxicity). According to these threshold values, five groups were defined: (i) lipopeptides with a high LPS neutralization, high bactericidal activity, and low toxicity (**BP389**, **BP473**, and **BP475**); (ii) lipopeptides with high bactericidal and LPS activity but highly toxic (**BP377**, **BP393**, and **BP490**); (iii) lipopeptides with high LPS neutralization but low bactericidal activity (**BP375**, **BP387**, **BP474**, **BP485**, and **BP500**); (iv) lipopeptides with high bactericidal activity but low LPS neutralization (**BP495** and **BP499**); and (v) lipopeptides with low bactericidal and LPS activity and low toxicity (**BP494**, **BP496**, **BP498**, **BP501**, **BP545**, **BP546**, **BP547**, **BP548**, **BP549**, and **BP550**). The best biological profile was considered for the peptides **BP389**, **BP473**, and **BP475**.

### Interaction between BP473-K(CF) and *X. fastidiosa*

**Fig 7:**
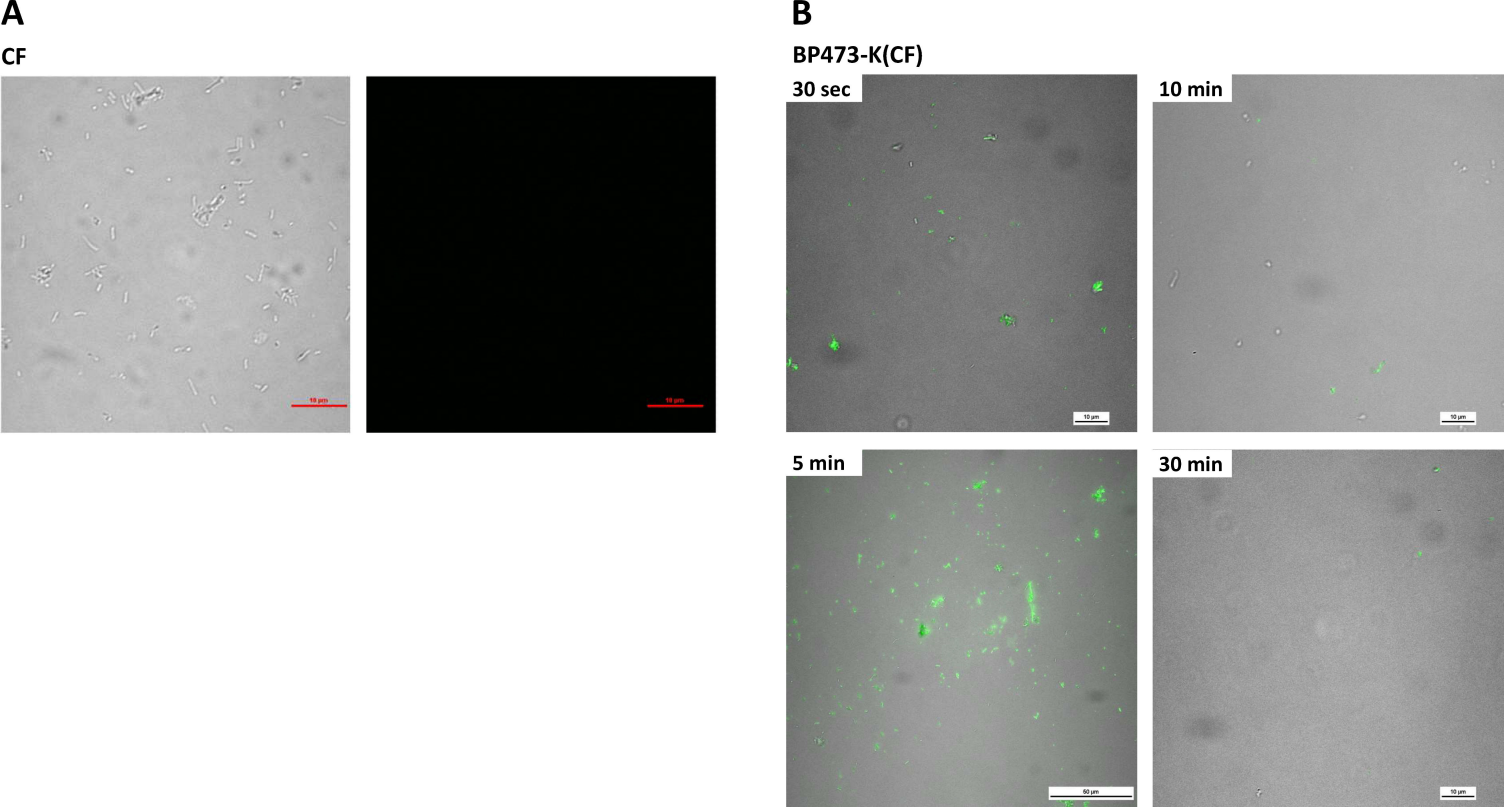
Microscopy images after a contact test with CF or **BP473-K(CF)** and *X. fastidiosa*. (**A**) On the right, confocal fluorescence microscopy channel (excitation at 492 nm and emission at 517 nm), and on the left, a merge of confocal and interference contrast channels. Image at 30 s contact test with CF and *X. fastidiosa*. Treatments were performed at 3.1 µM. (**B**) Merge of confocal and interference contrast channels. Images after different contact times with **BP473-K(CF)**. Treatments were performed at 5 µM.

The interaction between the labeled **BP473-K(CF)** and *X. fastidiosa* was studied using a contact test, and fixed samples were observed with confocal microscopy (Fig. 7). This experiment was also performed using CF alone for comparison purposes. After 30 s, cells treated with **BP473-K(CF)** at 3.1 µM showed a fluorescence signal, and no fluorescence was observed in cells treated with CF. After 5 min, fluorescence was detected for most of the cells treated with **BP473-K(CF)**, indicating a peptide-cell interaction. At longer contact times, there was a decrease in fluorescence, probably due to the lytic effect of the peptide.

## DISCUSSION

The use of peptides to control *X. fastidiosa* is a research topic that has begun to be explored in recent years, and several peptides with bactericidal effects against this bacterium have been identified (19, 20, 54). Among them, **BP178** is highlighted because, in addition to regulating various plant defense mechanisms, it also possesses bactericidal properties due to a lytic effect (19). The present work identified peptides able to interact with LPS of *X. fastidiosa*, which are a major virulence factor of this bacterium. These peptides were selected from previous libraries developed in our group with activity against other gram-negative plant pathogenic bacteria (24–26, 28, 48, 55).

The study of the interaction between peptides and LPS requires the use of a reliable method. Several techniques have previously been used to study the capacity of peptides to neutralize LPS, including surface plasmon resonance (56) or the detection of tumor necrosis factor-alpha in human monocytes using enzyme-linked immunosorbent assay techniques (57). In the present work, we used a *Limulus* amebocyte lysate assay because it is a simple and rapid method to detect LPS-protein interaction (58), thus permitting the screening of a large collection of peptides. This method was assayed and validated in the present study following the manufacturer’s recommendations with peptides **YW12D**, **LBP-14**, **Lf (28–34)**, and **BPI(84–99)**, which have been reported to reduce LPS-induced inflammation in mammal cells both *in vitro* and *in vivo* (33, 34, 59, 60).

We observed that the LPS fraction obtained from *X. fastidiosa* subsp. *fastidiosa IVIA 5387.2* cells was not pure enough in contrast to the commercial *E. coli* (O111) LPS. Additional stages of purification, like the hot-phenol method followed by dialysis, would provide LPS of greater purity (32, 61, 62), but they were not attempted in the present work. Fortunately, when the functionality of the *X. fastidiosa* extracts was checked, it was concluded that performing peptide-LPS neutralization assays with commercial *E. coli* endotoxins is equivalent to performing the test with *X. fastidiosa* LPS.

The activity of peptides on LPS neutralization revealed that the presence of a fatty acid chain in the cationic amphipathic peptide favors LPS-peptide interaction. In fact, among the 36 sequences tested, those with the highest capacity to neutralize LPS were cationic lipopeptides. Notably, six of these lipopeptides (**BP377**, **BP389**, **BP393**, **BP473**, **BP490**, and **BP500**) showed a ratio of reactive LPS close to zero. Similar to colistin and other polymyxin antibiotics (41, 42), these results suggested that there is an affinity between the positively charged moiety of peptides and the negatively charged phosphate groups of the lipid A, component of LPS. The hydrophobicity of the fatty acid chain might facilitate the interaction of the peptide sequence with the LPS. However, no correlation between the length or the position of the fatty acid chain and this activity could be found. In addition, the presence of a D-Phe at position 4 did not influence the interaction of the lipopeptide with LPS.

Among the six lipopeptides with a very high LPS neutralization level, five sequences (**BP377**, **BP389**, **BP393**, **BP473**, and **BP490**) also exhibited a high bactericidal activity against *X. fastidiosa*. Notably, **BP473** showed a reduction of cell viability >2.5 log at 12.5 µM. In addition, the bactericidal activity of this lipopeptide followed a dose-dependent relationship with a saturation pattern typical of bactericidal peptides (63). Confocal microscopy experiments confirmed the interaction and cell lytic effect of **BP473** because most *X. fastidiosa* cells showed fluorescence when treated with fluorescently labeled **BP473** at short contact test times, whereas a marked decrease in fluorescence was observed at longer times.

The biological profile of these lipopeptides indicates that the fatty acid chain may have a role in LPS neutralization and, in consequence, in the bactericidal activity against *X. fastidiosa*. It is known that the incorporation of a fatty acid into a peptide sequence clearly modifies its hydrophobicity, which facilitates its anchoring to the membranes of gram-negative bacteria, causing their disruption (64–66). Accordingly, in the case of colistin, after the electrostatic interaction with lipid A and subsequent insertion of the fatty acid chain, membrane disruption occurs (41, 42).

Interestingly, peptide **BP178** did not fit into the general relationship between bactericidal activity and LPS interaction and did not interact with LPS. This finding confirms that there are lytic peptides with high bactericidal activity that may act by different mechanisms to disturb the bacterial cell membrane and that are independent of LPS interaction, such as the case of the undecapeptide **BP100** (67) or the cyclic decapeptide **BPC194** (68).

**Fig 8:**
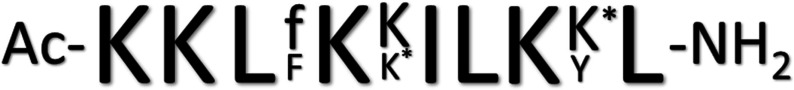
General sequence of lipopeptides with the best biological activity profile studied in this work: **BP389** (Phe^4^, Lys^6^, and Lys^10^[COC_3_H_7_]), **BP473** (D-Phe^4^, Lys^6^[COC_3_H_7_], and Tyr^10^), and **BP475** (D-Phe^4^, Lys^6^, and Lys^10^[COC_3_H_7_]). Lowercase letters correspond to a D-amino acid. Asterisk corresponds to a lysine incorporating a butanoyl group. Ac stands for acetyl.

Lipopeptides that exhibited the best biological activity profile in terms of high LPS neutralization, high bactericidal activity, and low toxicity were **BP389**, **BP473**, and **BP475** (Fig. 8). These peptides incorporate a butanoyl chain at positions 10, 6, and 10, respectively. In addition, **BP473** and **BP475** contain a D-Phe at position 4. Interestingly, these lipopeptides also stood up for their high antibacterial activity against other gram-negative plant pathogenic bacteria, including *Erwinia amylovora*, *Pseudomonas syringae* pv. syringae, *P. syringae* pv. actinidiae, *Xanthomonas arboricola* pv. pruni, *X. fragariae*, and *X. axonopodis* pv. vesicatoria (25, 26). Furthermore, these three lipopeptides were low hemolytic and phytotoxic (<10% hemolysis at 50 µM, leave lesion diameter ≤7 mm at 150 µM).

In summary, a collection of lipopeptides with the capacity to neutralize LPS was identified. Notably, those with the highest LPS neutralization were also highly active against *X. fastidiosa* and low toxic. Therefore, these compounds constitute promising candidates to mitigate infections caused by *X. fastidiosa* in host plants. Studies to decipher structural-activity relationships and their mechanism of action are ongoing.

## SUPPLEMENTAL MATERIAL

Supplemental material aem.00734-25-s0001.pdf Analysis and characterization data of peptides; biological activity of peptides; and HPLC, ESI-MS, and HRMS of purified lipopeptides.

ASM does not own the copyrights to Supplemental Material that may be linked to, or accessed through, an article. The authors have granted ASM a non-exclusive, world-wide license to publish the Supplemental Material files. Please contact the corresponding author directly for reuse.
