# Supplementary material for: Synthetic lipopeptides that interact with lipopolysaccharides are potent bactericidal compounds against Xylella fastidiosa
Source: Appl Environ Microbiol. 2025 Jul 30;91(8):e00734-25. doi: 10.1128/aem.00734-25 (PMC12366332; doi:10.1128/aem.00734-25)
Supplement: Supplemental material — Analysis and characterization data of peptides; biological activity of peptides; and HPLC, ESI-MS, and HRMS of purified lipopeptides. [file aem.00734-25-s0001.pdf]

SUPPLEMENTARY MATERIAL

Synthetic lipopeptides that interact with lipopolysaccharides are potent bactericidal compounds against *Xylella fastidiosa*

Pau Caravaca-Fuentes[1,2], Laura Montesinos[1], Jordi Lamata[2], Marta Planas[2], Lidia Feliu[2,#], Emilio Montesinos[1,#]

[1] Laboratory of Plant Pathology (CIDSAV), Institute of Agrifood Technology, University of Girona, Girona, Spain.

[2] LIPPSO, Chemistry Department, Institute of Agrifood Technology, University of Girona, Girona, Spain

Running Head: Synthetic lipopeptides against *Xylella fastidiosa*

Table of contents

1. Analysis and characterization data of peptides 2

2. Biological activity of peptides 4

3. HPLC, ESI-MS and HRMS of purified lipopeptides........................... 9

1. Analysis and characterization data of peptides

Table S1. Sequences, retention time, purity, and mass spectrometry data of peptides

Code	Sequence[1]	t_R (min)[2]	Purity (%)[3]	HRMS (ESI)		
				Molecular formula	Calcd	Found[4]
**Linear undecapeptides (CECMEL11)**						
**BP013**	FKLFKKILKVL-NH_2	5,48	>99	$C_{71}H_{122}N_{16}O_{11}$ $[M + H]^+$	-	1375,8*
**BP015**	KKLFKKILKVL-NH_2	4,89	82	$C_{68}H_{125}N_{17}O_{11}$ $[M + H]^+$	-	1356,0*
**BP016**	KKLFKKILKKL-NH_2	5,98	>99	$C_{69}H_{128}N_{18}O_{11}$$[M + 3H]^{3+}$	462,6743	462,6733
**BP022**	Ac-LKLFKKILKVL-NH_2	5,05	>99	$C_{70}H_{126}N_{16}O_{12}$ $[M + H]^+$	-	1384,9*
**BP076**	KKLFKKILKFL-NH_2	5,12	>99	$C_{72}H_{126}N_{17}O_{11}$ $[M + H]^+$	-	1405,1*
**BP100**	KKLFKKILKYL-NH_2	5,32	>99	$C_{72}H_{127}N_{17}O_{12}$ $[M+2H]^{2+}$	843,0230	843,0206
**Linear undecapeptides derived from BP100 containing a D-amino acid**						
**BP143**	KKLfKKILKYL-NH_2	6,28	84	$C_{72}H_{125}N_{17}O_{12}$ $[M + H]^+$	-	1421,0*
**BP144**	KKlFKKILKYL-NH_2	5,07	85	$C_{72}H_{125}N_{17}O_{12}$ $[M + H]^+$	-	1421,1*
**BP145**	KkLFKKILKYL-NH_2	5,14	>99	$C_{72}H_{125}N_{17}O_{12}$ $[M + H]^+$	-	1421,1*
**BP146**	KKLFKkILKYL-NH_2	4,90	80	$C_{72}H_{125}N_{17}O_{12}$ $[M + H]^+$	-	1421,1*
**Lipopeptides derived from BP100**						
**BP375**	Ac-KKLFKKIK(COC_5H_{11})KYL-NH_2	7,17	>99	$C_{80}H_{140}N_{18}O_{14}$ $[M + 2H]^{2+}$	788,5393	788,5383
**BP377**	Ac-KKLFKKILKK(COC_5H_{11})L-NH_2	7,58	>99	$C_{77}H_{142}N_{18}O_{13}$ $[M + 2H]^{2+}$	763,5496	763,5486
**BP387**	Ac-KKLFKKIK(COC_3H_7)KYL-NH_2	6,49	>99	$C_{78}H_{137}N_{18}O_{14}$ $[M + 3H]^{3+}$	516,6848	516,6839
**BP389**	Ac-KKLFKKILKK(COC_3H_7)L-NH_2	6,83	>99	$C_{75}H_{138}N_{18}O_{13}$ $[M + 2H]^{2+}$	749,5340	749,5328
**BP393**	Ac-KK($COC_{11}H_{23}$)LFKKILKYL-NH_2	7,68	>99	$C_{86}H_{151}N_{17}O_{14}$ $[M + 2H]^{2+}$	823,0808	823,0789
**BP473**	Ac-KKLfKK(COC_3H_7)ILKYL-NH_2	5,99	>99	$C_{78}H_{134}N_{17}O_{14}$ [M + H]+	1533,0291	1533,024
**BP474**	Ac-KKLfKKIK(COC_3H_7)KYL-NH_2	5,36	>99	$C_{78}H_{135}N_{18}O_{14}$ $[M + H]^+$	1548,0400	1548,0381
**BP475**	Ac-KKLfKKILKK(COC_3H_7)L-NH_2	5,69	>99	$C_{75}H_{136}N_{18}O_{13}Na$ $[M + Na]^+$	1520,0426	1520,0424
**BP485**	C_3H_7CO-KKLfKKILKYL-NH_2	6,11	>99	$C_{76}H_{132}N_{17}O_{13}$ $[M + H]^+$	1491,0185	1491,0154
**BP490**	Ac-KKLfKKIK($COC_{11}H_{23}$)KYL-NH_2	7,12	>99	$C_{86}H_{151}N_{18}O_{14}$ $[M + H]^+$	1660,1652	1660,1635
**BP494**	Ac-KKLfKKK(COC_5H_{11})LKYL-NH_2	5,42	>99	$C_{80}H_{139}N_{18}O_{14}$ $[M + H]^+$	1576,0713	1576,0683
**BP495**	Ac-KKLfKKILKYK(COC_5H_{11})-NH_2	5,44	>99	$C_{80}H_{139}N_{18}O_{14}$ $[M + H]^+$	1576,0713	1576,0683
**BP496**	Ac-KKLfKKILKYK(COC_3H_7)-NH_2	5,11	>99	$C_{86}H_{135}N_{18}O_{14}$ $[M + H]^+$	1548,0400	1548,0367
**BP498**	Ac-KKLfK(COC_3H_7)KILKYL-NH_2	6,19	>99	$C_{78}H_{134}N_{17}O_{14}$ $[M + H]^+$	1533,0291	1533,0225
**BP499**	Ac-KKLfKKILK(COC_3H_7)YL-NH_2	5,80	>99	$C_{78}H_{134}N_{17}O_{14}$ $[M + H]^+$	1533,0291	1533,0269
**BP500**	Ac-KKK($COC_{11}H_{23}$)fKKILKYL-NH_2	6,80	>99	$C_{86}H_{151}N_{18}O_{14}$ $[M + H]^+$	1661,1684	1661,1667
**BP501**	Ac-KKLHKKILKK(COC_3H_7)L-NH_2	4,63	>99	$C_{72}H_{135}N_{20}O_{13}$ $[M + H]^+$	1488,0512	1488,0511
**BP545**	Ac-K(COC_3H_7)KLfKKILKYL-NH_2	6,44	>99	$C_{78}H_{134}N_{17}O_{14}$ $[M + H]^+$	1533,0291	1533,0259
**BP546**	Ac-KKK(COC_3H_7)fKKKLKYL-NH_2	4,57	>99	$C_{78}H_{136}N_{19}O_{14}$ $[M + H]^+$	1563,0509	1563,0501

**BP547**	Ac-KKLk(COC_3H_7)KKILIYL-NH_2	5,50	91	$C_{75}H_{136}N_{17}O_{14}$ [M + H]$^+$	1499,0447	1499,0438
**BP548**	Ac-KKLfKKK(COC_3H_7)LKYL-NH_2	5,26	>99	$C_{78}H_{135}N_{18}O_{14}$ [M + H]$^+$	1548,0400	1548,0372
**BP549**	Ac-KKK(COC_3H_7)fKKILKYL-NH_2	5,47	>99	$C_{78}H_{135}N_{18}O_{14}$ [M + H]$^+$	1548,0400	1548,0408
**BP550**	Ac-KKLk(COC_3H_7)KKILKYL-NH_2	5,23	>99	$C_{75}H_{137}N_{18}O_{14}$ [M + H]$^+$	1514,0556	1514,0547
**Cyclic decapeptide (CYCLO10)**						
**BPC098W**	c(LLKKKWKKLQ)	6,07	>99	$C_{64}H_{111}N_{17}O_{11}$ [M + H]$^+$	1294,8722	1294,8714
**Peptide analogue of KSL-W**						
**BP442**	KKVVFWVKFk-NH_2	4,75	>99	$C_{68}H_{106}N_{16}$O10 [M + H]$^+$	1307,8272	1307,8342
**Peptide conjugate derived from BP100**						
**BP178**	KKLFKKILKYLAGPAGIGKFLHSAKKDEL-OH	4,77	>99	$C_{155}H_{257}N_{39}O_{36}$ [M+2H]$^{2+}$	1621,4812	1621,4809
**Peptides described with LPS-neutralizing activity**						
**BPI(84-99)**	NIKISGKWKAQKRFLK-NH_2	4,98	>99	$C_{79}H_{123}N_{25}O_{16}$ [M + H]$^+$	1678,9652	1678,9615
**LBP-14**	RVQGRWKVRASFFK-NH_2	4,78	>99	$C_{94}H_{161}N_{31}O_{19}$ [M + H]$^+$	2029,2658	2029,2658
**Lf(28-34)**	RKVRGPP-NH_2	3,11	>99	$C_{35}H_{65}N_{15}O_7$ [M + H]$^+$	808,5264	808,5275
**YW12D**	YVKLWRMIKFIR-NH_2	5,63	>99	$C_{81}H_{130}N_{22}O_{13}S$ [M + H]$^+$	1651,9981	1651,9966
**CF-labeled BP473 analogues**						
**BP473-CF**	CF-KKLfKK(COC_3H_7)ILKYL-NH_2	7,00	>99	$C_{97}H_{141}N_{17}O_{19}$ [M + H]$^+$	1849,0662	1849,0623
**BP473-K(CF)**	Ac-K(CF)KKLfKK(COC_3H_7)ILKYL-NH_2	6,58	>99	$C_{105}H_{155}N_{19}O_{21}$ [M + H]$^+$	2019,1718	2019,1681

[1]Lowercase letters indicate a D-amino acid

[2]HPLC retention time

[3]Percentage determined by HPLC at 220 nm

[4]The asterisk indicates that results correspond to ESI-MS analysis

2. Biological activity of peptides

Table S2. LPS neutralization and bactericidal activity of peptides

Code	LPS neutralization[1]			Bactericidal activity[2]						
				50 µM			12.5 µM		3.1 µM	
	Ratio (treatment/NTC)		Level	Reduction in viability (Log N_0/N)		Level	Reduction in viability (Log N_0/N)		Reduction in viability (Log N_0/N)	
**Linear undecapeptides (CECMEL11)**										
**BP013**	1,13 ± 0,02	rs	Low	1,03 ± 0,12	ghij	Low				
**BP015**	1,10 ± 0,02	rs	Low	1,13 ± 0,13	ijk	Low				
**BP016**	1,13 ± 0,06	s	Low	0,00 ± 0,10	a	Low				
**BP022**	0,61 ± 0,01	k	Moderate	**1,87** ± 0,03	no	Moderate				
**BP076**	0,36 ± 0,04	hi	Moderate	0,91 ± 0,11	fghi	Low				
**BP100**	0,34 ± 0,02	h	Moderate	1,92 ± 0,09	nop	Moderate				
**Linear undecapeptides derived from BP100 containing a D-amino acid**										
**BP143**	0,70 ± 0,06	lm	Moderate	0,89 ± 0,10	fgh	Low				
**BP144**	1,07 ± 0,05	r	Low	0,68 ± 0,08	def	Low				
**BP145**	0,76 ± 0,03	n	Low	1,08 ± 0,22	hij	Low				
**BP146**	0,73 ± 0,02	mn	Low	1,25 ± 0,13	jk	Low				
**Lipopeptides derived from BP100**										
**BP375**	0,20 ± 0,01	fe	High	1,37 ± 0,12	kl	Low				
**BP377**	0,13 ± 0,03	cde	Very High	**2,71** ± 0,04	s	Very High	**1,88** ± 0,06	e	1,34 ± 0,06	c
**BP387**	0,17 ± 0,02	ef	High	1,96 ± 0,09	nop	Moderate	0,83 ± 0,06	c		
**BP389**	0,03 ± 0,00	a	Very High	**2,41** ± 0,05	r	High	**1,51** ± 0,10	d	0,02 ± 0,07	a
**BP393**	0,03 ± 0,01	ab	Very High	**2,25** ± 0,11	qr	High	0,00 ± 0,28	a	0,01 ± 0,16	a
**BP400**				1,06 ± 0,08		Low				
**BP410**	0,01 ± 0,01	a	High	1,35 ± 0,06	jk	Low				

Table S2 (continuation). LPS neutralization and bactericidal activity of peptides

Code	LPS neutralization[1]			Bactericidal Activity[2]						
				50 µM			12.5 µM		3.1 µM	
	Ratio (treatment/NTC)		Level	Reduction in viability (Log N_0/N)		Level	Reduction in viability (Log N_0/N)		Reduction in viability (Log N_0/N)	
BP473	0,11 ± 0,01	cd	Very High	**2,63** ± 0,11	s	Very High	**2,67** ± 0,05	h	0,64 ± 0,11	b
BP474	0,16 ± 0,01	def	High	1,76 ± 0,28	mn	Moderate				
BP475	0,24 ± 0,04	g	High	**2,34** ± 0,05	r	High	1,04 ± 0,25	c		
BP485	0,18 ± 0,01	f	High	1,92 ± 0,23	nop	Moderate	0,49 ± 0,15	b		
BP490	0,09 ± 0,02	bc	Very High	**2,35** ± 0,06	r	High	**2,06** ± 0,14	ef	1,28 ± 0,07	c
BP494	0,51 ± 0,02	j	Moderate	1,21 ± 0,30	jk	Low				
BP495	0,55 ± 0,03	l	Moderate	**2,26** ± 0,16	qr	High	0,03 ± 0,10	a		
BP496	0,92 ± 0,05	p	Low	0,69 ± 0,06	def	Low				
BP498	0,40 ± 0,05	i	Moderate	1,58 ± 0,14	lm	Moderate				
BP499	0,34 ± 0,02	h	Moderate	**2,11** ± 0,17	pq	High	**2,27** ± 0,26	g	0,63 0,09	b
BP500	0,04 ± 0,01	ab	Very High	1,37 ± 0,14	kl	Low				
BP501	0,71 ± 0,03	op	Low	0,30 ± 0,07	bc	Low				
BP545	0,56 ± 0,06	j	Moderate	**2,00** ± 0,16	op	High	0,54 ± 0,06	b		
BP546	0,73 ± 0,10	mn	Low	0,34 ± 0,24	bc	Low				
BP547	0,99 ± 0,05	q	Low	0,05 ± 0,31	a	Low				
BP548	0,65 ± 0,02	kl	Moderate	0,28 ± 0,07	b	Low				
BP549	1,10 ± 0,02	rs	Low	1,19 ± 0,14	jk	Low				
BP550	1,09 ± 0,02	rs	Low	0,00 ± 0,02	a	Low				
**Cyclic decapeptide (CYCLO10)**										
BPC098W	0,83 ± 0,05	o	Low	0,78 ± 0,20	ef	Low				

Table S2 (continuation). LPS neutralization and bactericidal activity of peptides

Code	LPS neutralization[1]		Bactericidal Activity[2]			
			50 µM		12.5 µM	3.1 µM
	Ratio (treatment/NTC)	Level	Reduction in viability (Log N_0/N)	Level	Reduction in viability (Log N_0/N)	Reduction in viability (Log N_0/N)
**Peptide analogue of KSL-W**						
**BP442**	0,76 ± 0,02 n	**Low**	1,09 ± 0,18 **hij**	**Low**		
**Peptide conjugate derived from BP100**						
**BP178**	1,00 ± 0,03 rs	**Low**	**2,60 ± 0,17** s	**Very High**		
**Peptides described with LPS-neutralizing activity**						
**BPI(84-99)**	0,29 ± 0,02	**Moderate**	0,84 ± 0,09	**Low**		
**LBP-14**	0,23 ± 0,03	**High**	0,51 ± 0,13	**Low**		
**Lf(28-34)**	0,30 ± 0,05	**Moderate**	0,00 ± 0,08	**Low**		
**YW12D**	0,21 ± 0,03	**High**	0,59 ± 0,06	**Low**		
**CF-labeled BP473 analogues[3]**						
**BP473-CF**	nd		0,91 ± 0,12			
**BP473-K(CF)**	nd		1,78 ± 0,05			

[1]LPS neutralization is represented as the ratio between OD_{405} of treatment and of a non-treated control. Peptides were tested at 150 µM. The confidence interval is shown (α =0.05). Values sharing the same letters are not significantly different according to the Duncan's test (p<0.05).

[2]Reduction in viability of *X. fastidiosa* subsp. *fastidiosa* IVIA5387.2, ($LogN_0$/N), where N_0 is 10^7 CFU/ml in a non-treated control and N is CFU/ml of the treatment. Values are the means of three replicates. Peptides where tested at concentrations indicated in the column, except for reference peptide **BP178** which was tested at 25 µM. The confidence interval is shown (α =0,05). Values sharing the same letters are not significantly different according to the Duncan's test (p<0.05).

[3] The bactericidal activity of CF-labeled **BP473** analogues was tested at 25 µM.

Table S3. Hemolytic activity and size of the lesion in infiltrated tobacco leaves

Code	Hemolytic activity (%)[1]	Phytotoxicity (mm)[2]
	50 µM	150 µM
**Melittin**	100 ± 0	10 ± 3
**BP375**	23 ± 3	10 ± 1
**BP377**	71 ± 2	11 ± 1
**BP387**	4 ± 1	8 ± 1
**BP389**	9 ± 4	7 ± 1
**BP393**	93 ± 2	6 ± 1
**BP473**	7 ± 3	4 ± 1
**BP474**	0 ± 0	2 ± 2
**BP475**	0 ± 0	4 ± 1
**BP485**	3 ± 0	3 ± 1
**BP490**	100 ± 3	6 ± 2
**BP494**	0 ± 1	6 ± 1
**BP495**	0 ± 0	6 ± 2
**BP496**	0 ± 1	4 ± 1
**BP498**	9 ± 1	7 ± 3
**BP499**	2 ± 1	7 ± 2
**BP500**	99 ± 2	8 ± 2
**BP501**	2 ± 1	0 ± 0
**BP545**	5 ± 0	4 ± 2
**BP546**	0 ± 0	0 ± 0
**BP547**	0 ± 0	2 ± 2
**BP548**	0 ± 0	6 ± 1
**BP549**	1 ± 0	5 ± 1
**BP550**	3 ± 1	5 ± 1
**YW12D**	12 ± 3	1 ± 0
**LBP-14**	7 ± 2	1 ± 0
**Lf(28-34)**	1 ± 1	0 ± 0
**BPI(84-99)**	7 ± 1	1 ± 0

[1]Percent hemolysis at 50 µM plus confidence interval (α=0.05).

[2]Diameter of the lesion (mm) at 150 M plus confidence interval (α=0.05).

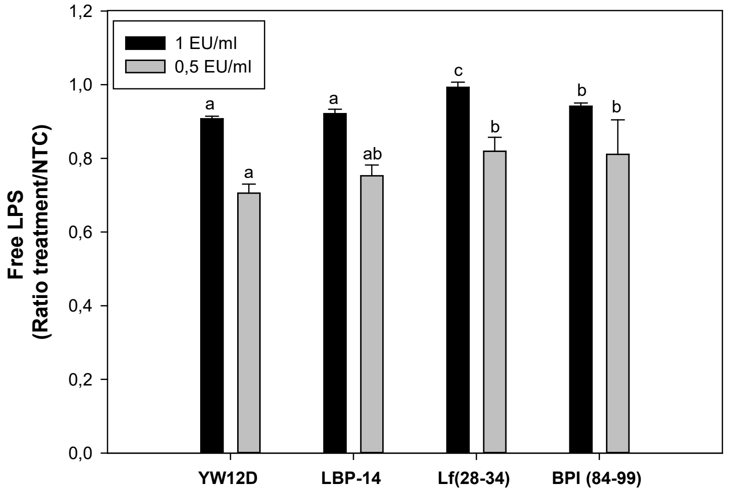

Figure S1. LPS neutralization test of peptides with previously described LPS interaction at 25 µM, using lyophilized *E. coli* 0111:B4 LPS at 1 EU/ml (black bars) and 0.5 EU/ml (grey bars). Ratio of OD at 405 nm of treatment and non-treated control is represented. Values are the means of three replicates and error bars represent confidence interval (α=0.05). For every LPS concentration, compounds sharing the same letters are not significantly different according to the Duncan's test ($p < 0.05$)

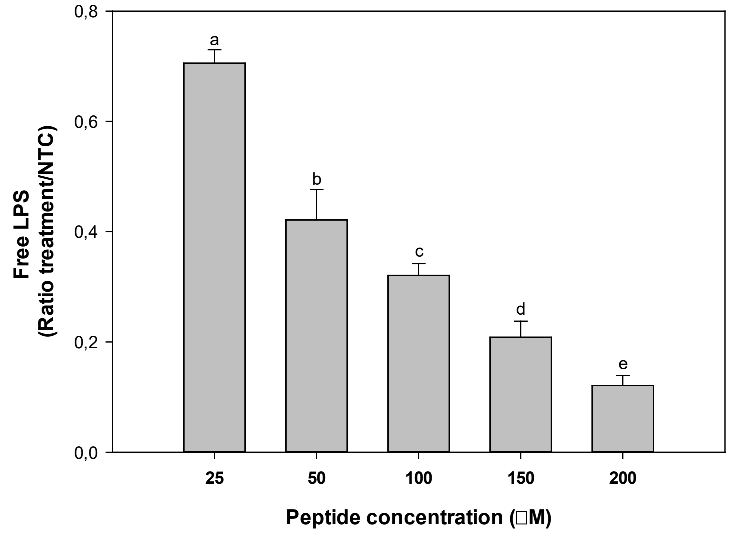

Figure S2. LPS neutralization test for the reference peptide **YW12D** at different concentrations. Bars correspond to ratio between treatment and non-treated control with lyophilized *E. coli* 0111:B4 LPS at 0.5 EU/ml. Values are the means of three replicates and error bars represent confidence interval (α=0.05). Concentrations sharing the same letters are not significantly different according to the Duncan's test ($p < 0.05$)

3. HPLC, ESI-MS and HRMS of purified lipopeptides

Ac-Lys-Lys-Leu-His-Lys-Lys-Ile-Leu-Lys-Lys(COC_3H_7)-Leu-NH_2 (BP501)

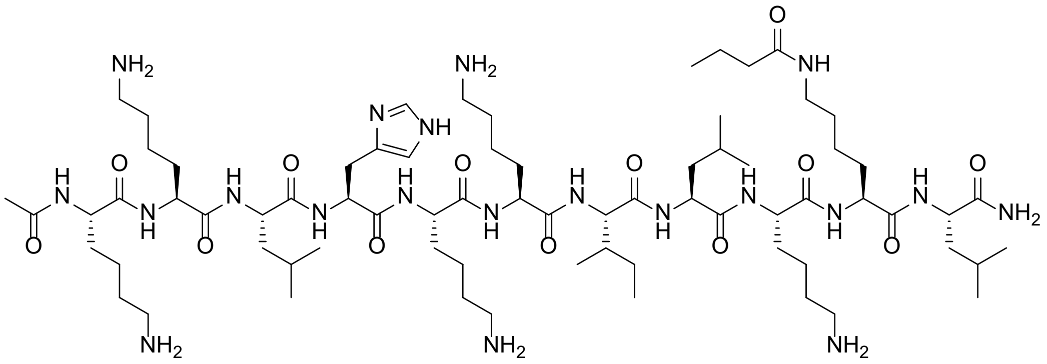

HPLC of purified peptide (λ=220 nm)

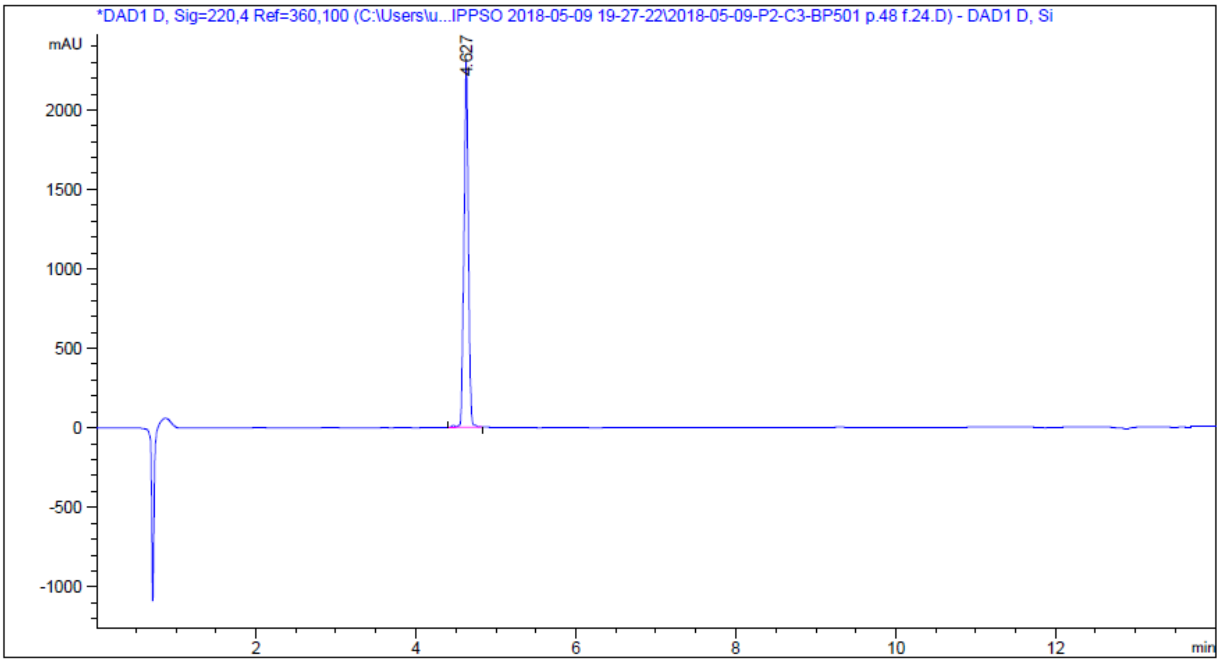

Peak #	RetTime [min]	Type	Width [min]	Area [mAU*s]	Height [mAU]	Area %
1	4.627	VV R	0.0567	8830.34766	2305.12036	100.0000

ESI-MS (*m/z*)

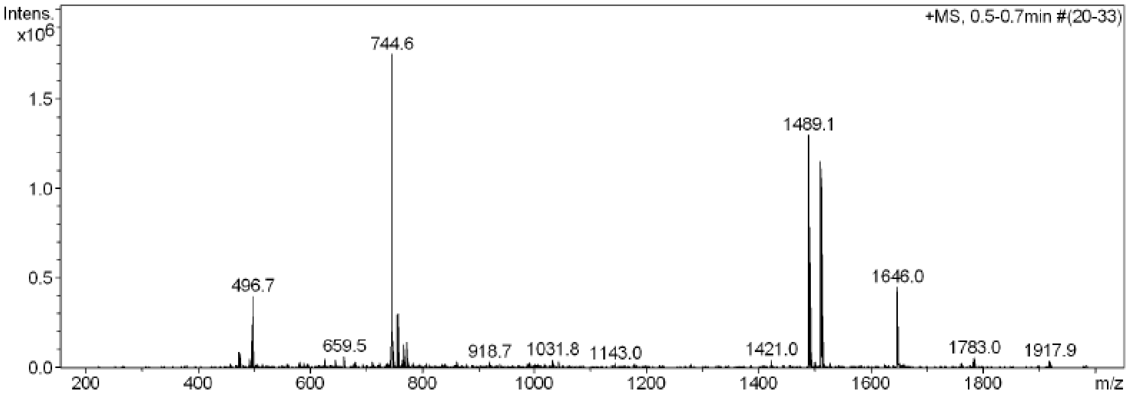

HRMS (*m/z*)

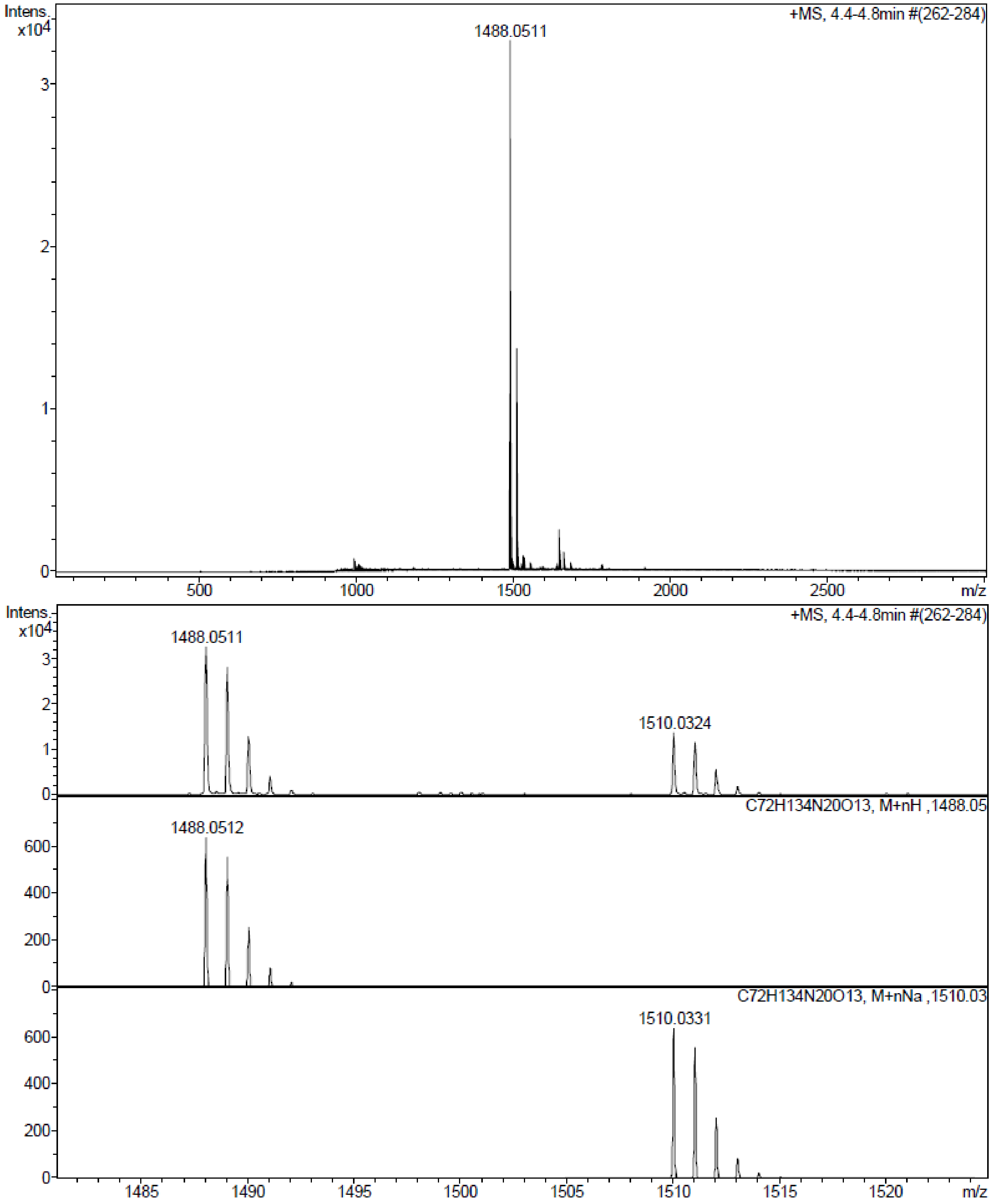

Intens.
x10^4
+MS, 4.4-4.8min #(262-284)
1488.0511
3
2
1
0
500
1000
1500
2000
2500
m/z
Intens.
x10^4
+MS, 4.4-4.8min #(262-284)
1488.0511
1510.0324
C72H134N20O13, M+nH ,1488.05
1488.0512
600
400
200
C72H134N20O13, M+nNa ,1510.03
1510.0331
1485
1490
1495
1500
1505
1510
1515
1520
m/z

Ac-Lys(COC_3H_7)-Lys-Leu-D-Phe-Lys-Lys-Ile-Leu-Lys-Tyr-Leu-NH_2 (BP545)

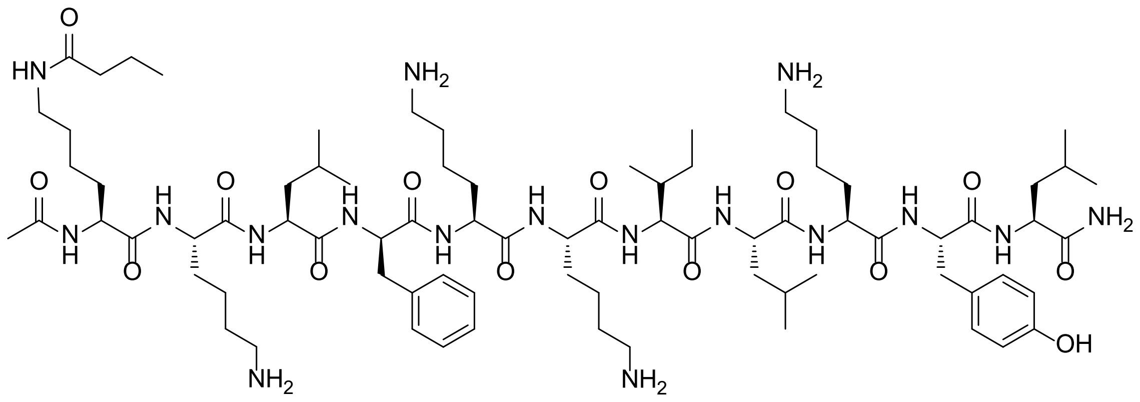

HPLC of purified peptide (λ=220 nm)

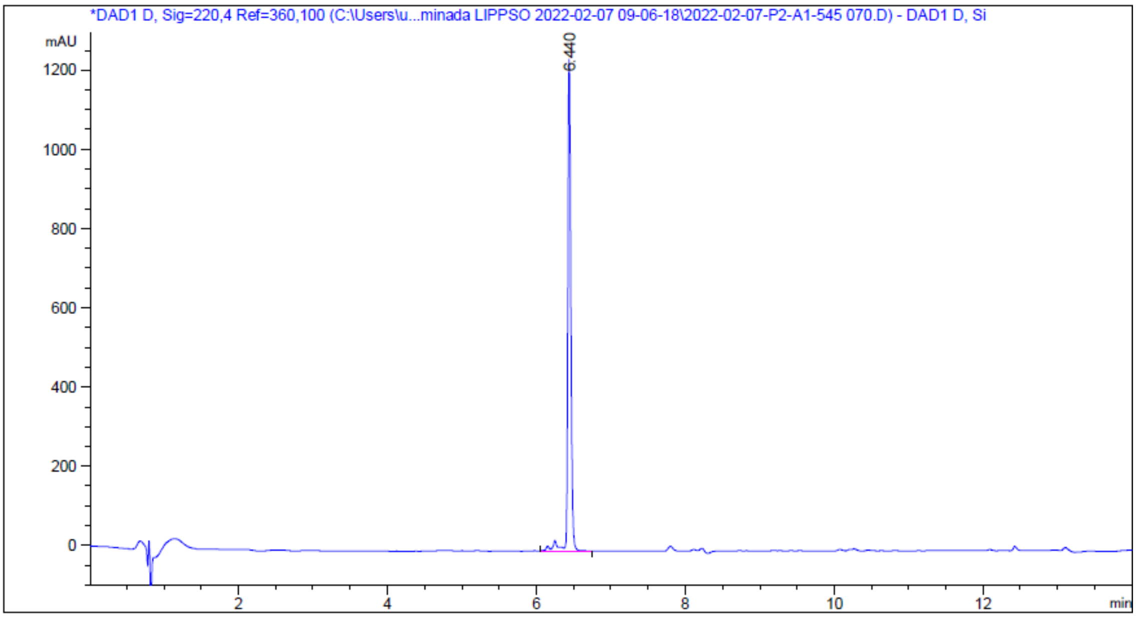

Peak #	RetTime [min]	Type	Width [min]	Area [mAU*s]	Height [mAU]	Area %
1	6.440	VB R	0.0433	3550.27856	1242.00403	100.0000

ESI-MS (*m/z*)

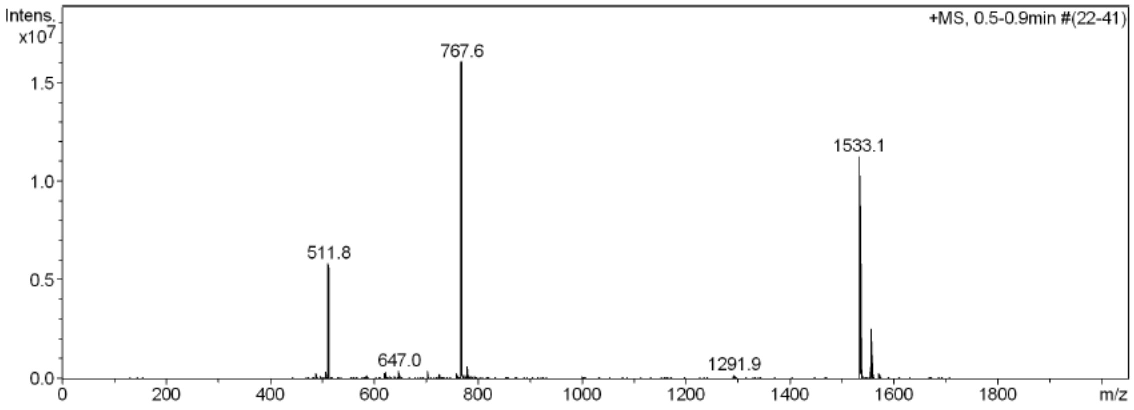

HRMS (m/z)

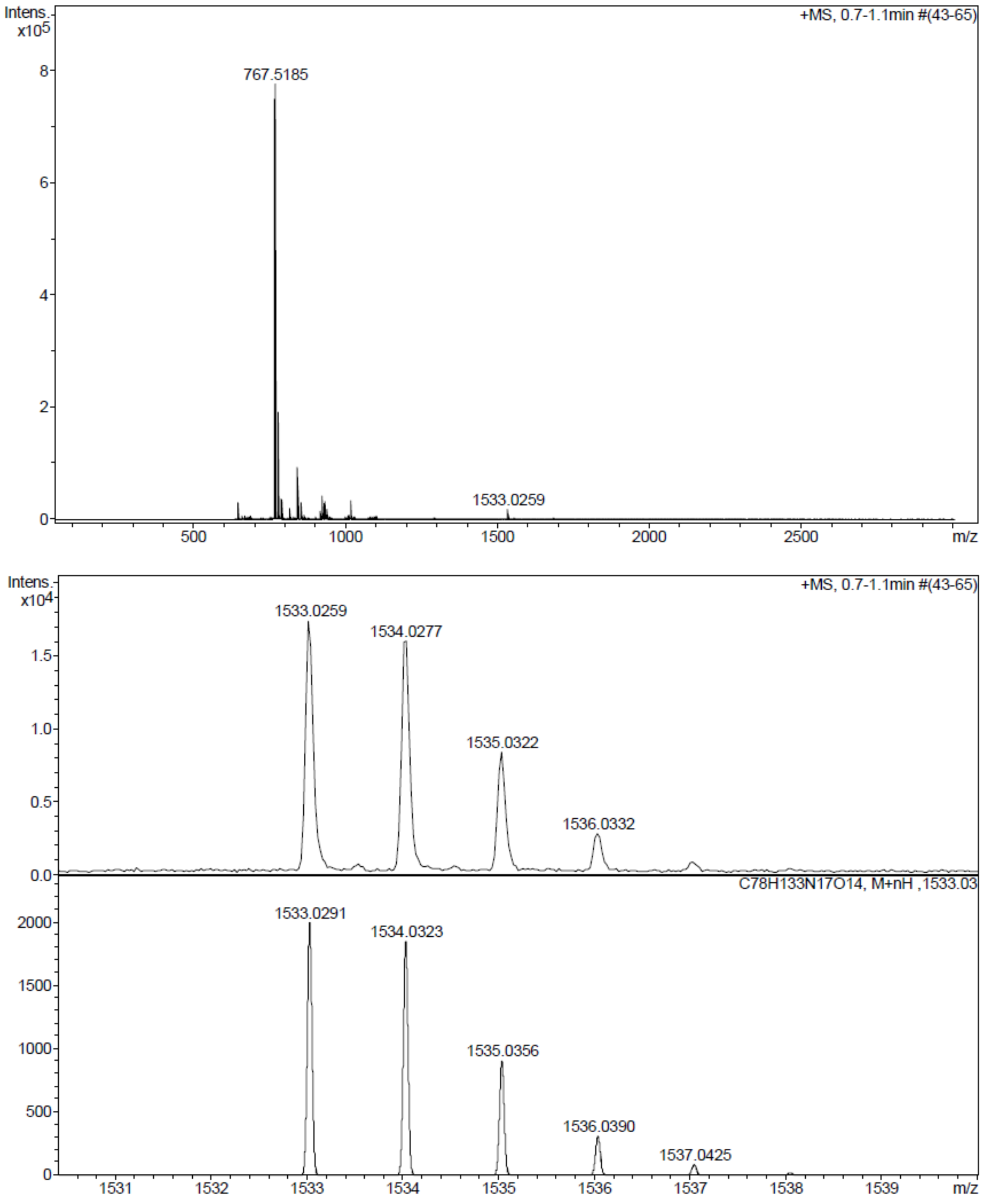
Intens.
x10^5
+MS, 0.7-1.1min #(43-65)
767.5185
1533.0259
m/z
Intens.
x10^4
+MS, 0.7-1.1min #(43-65)
1533.0259
1534.0277
1535.0322
1536.0332
C78H133N17O14, M+nH ,1533.03
1533.0291
1534.0323
1535.0356
1536.0390
1537.0425
m/z

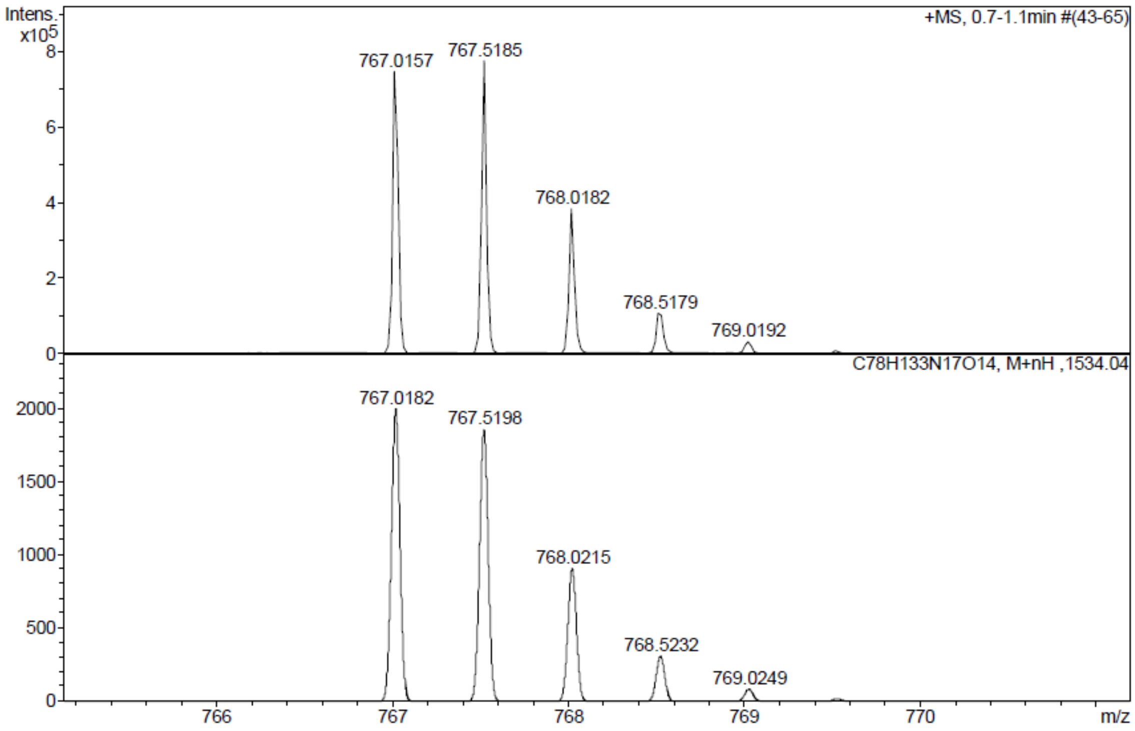
Intens.
x10^5
+MS, 0.7-1.1min #(43-65)
767.0157
767.5185
768.0182
768.5179
769.0192
C78H133N17O14, M+nH ,1534.04
767.0182
767.5198
768.0215
768.5232
769.0249
m/z

Ac-Lys-Lys-Lys(COC_3H_7)-D-Phe-Lys-Lys-Lys-Leu-Lys-Tyr-Leu-NH_2 (BP546)

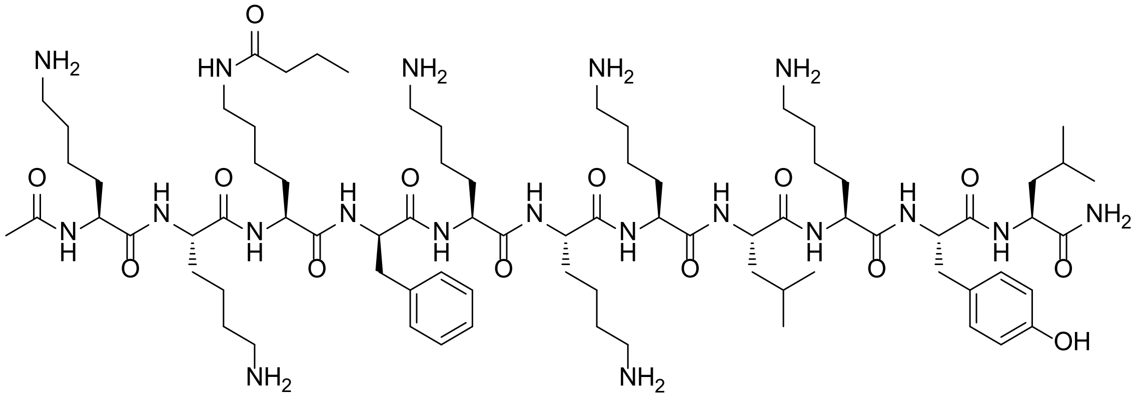

HPLC of purified peptide (λ=220 nm)

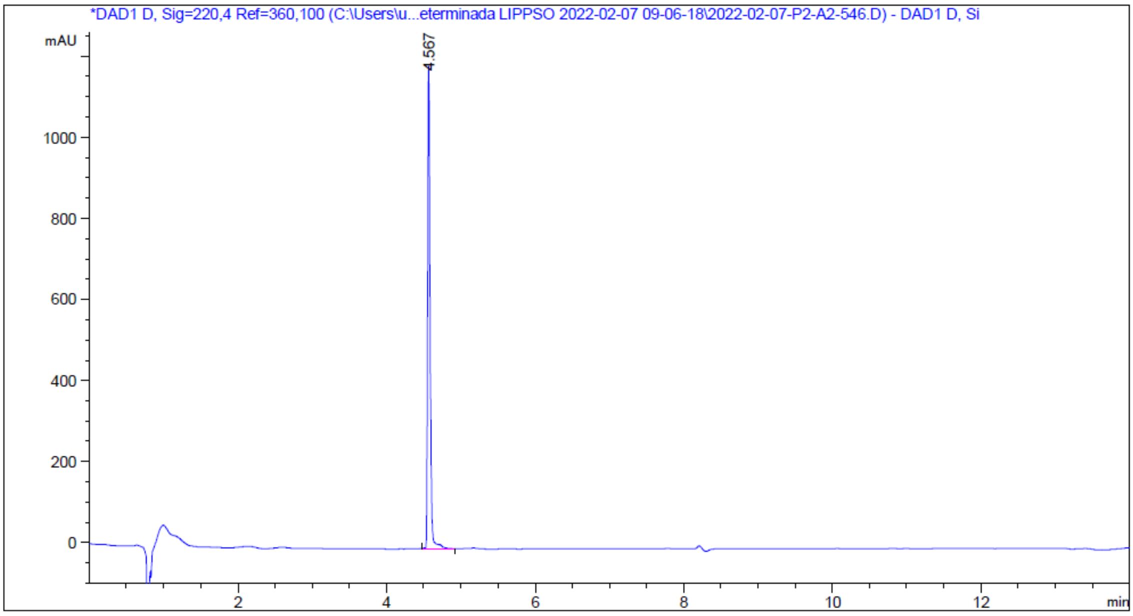

Peak #	RetTime [min]	Type	Width [min]	Area [mAU*s]	Height [mAU]	Area %
1	4.567	VV R	0.0365	2818.58154	1193.75403	100.0000

ESI-MS (*m/z*)

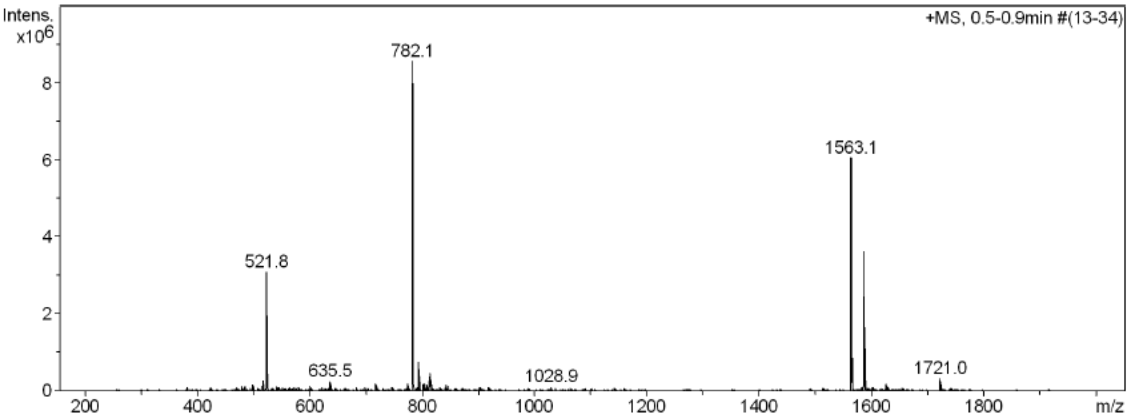

HRMS (*m/z*)

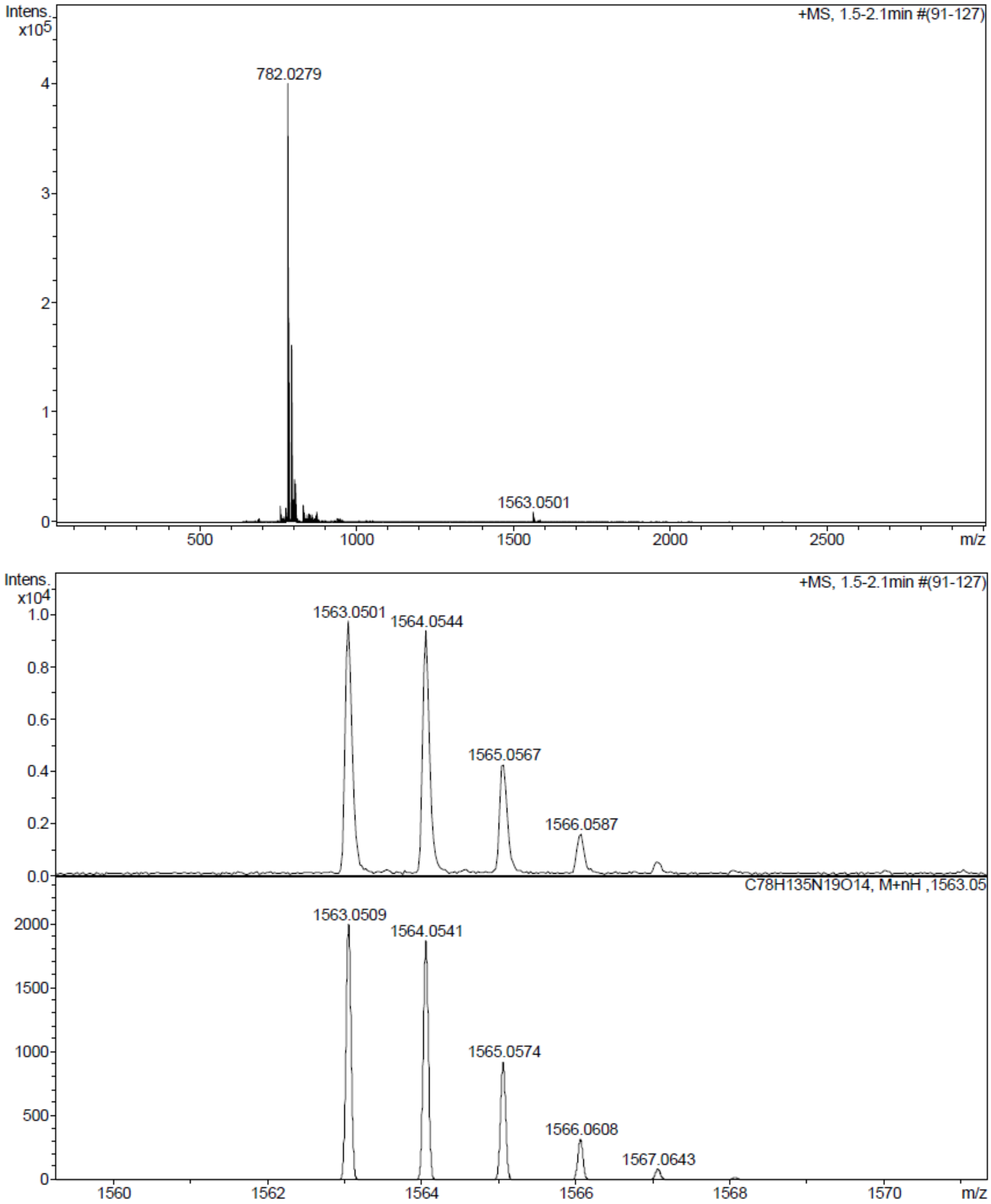
Intens.
x10^5
+MS, 1.5-2.1min #(91-127)
782.0279
1563.0501
m/z
Intens.
x10^4
+MS, 1.5-2.1min #(91-127)
1563.0501
1564.0544
1565.0567
1566.0587
C78H135N19O14, M+nH ,1563.05
1563.0509
1564.0541
1565.0574
1566.0608
1567.0643
m/z

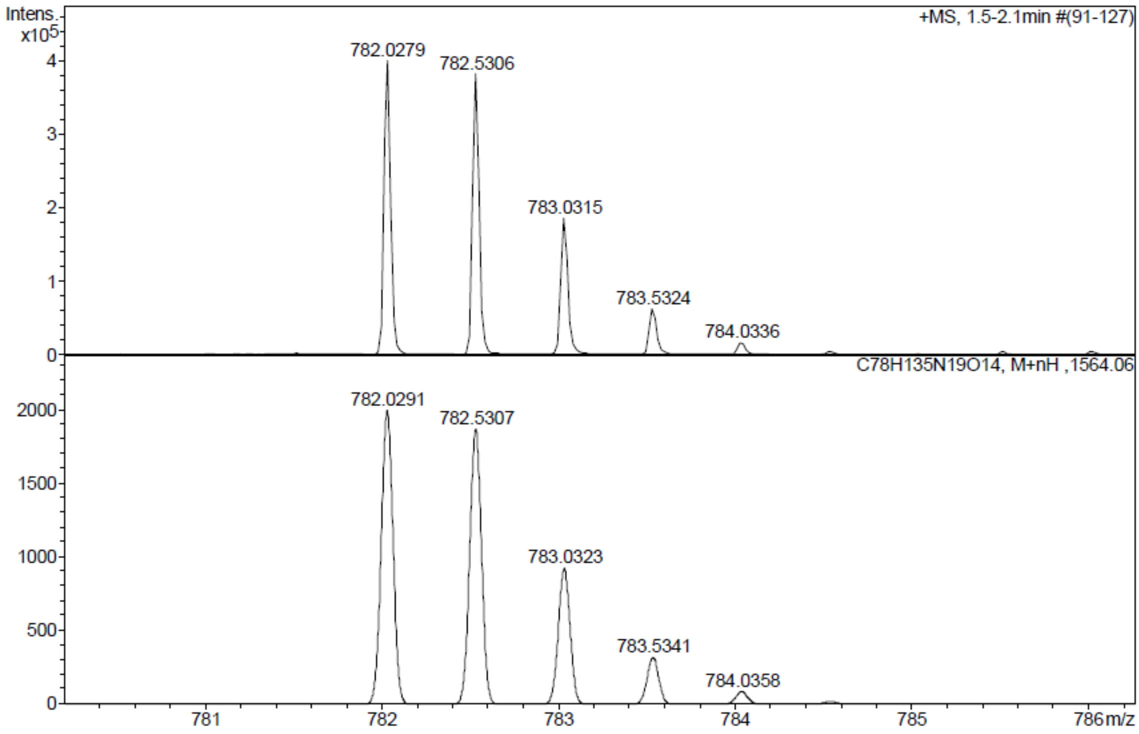

Intens.
x10^5
+MS, 1.5-2.1min #(91-127)
782.0279
782.5306
783.0315
783.5324
784.0336
C78H135N19O14, M+nH ,1564.06
782.0291
782.5307
783.0323
783.5341
784.0358
m/z

Ac-Lys-Lys-Leu-D-Lys(COC_3H_7)-Lys-Lys-Ile-Leu-Ile-Tyr-Leu-NH_2 (BP547)

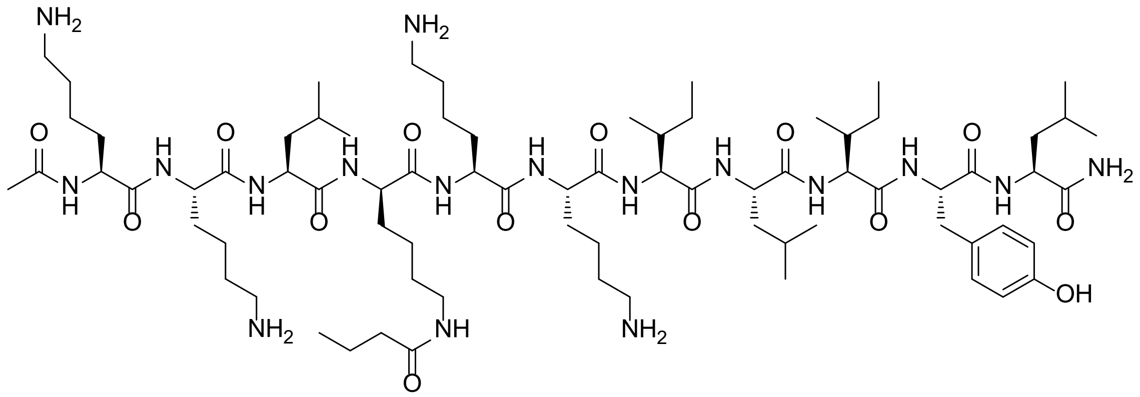

HPLC of purified peptide (λ=220 nm)

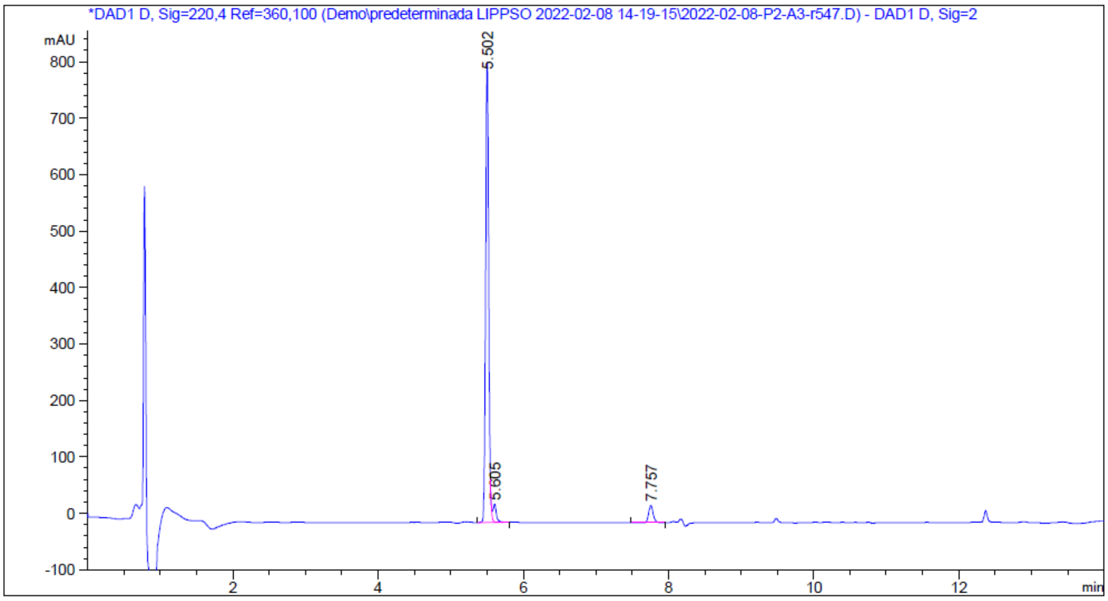

Peak #	RetTime [min]	Type	Width [min]	Area [mAU*s]	Height [mAU]	Area %
1	5.502	BV R	0.0441	2390.14844	818.49677	90.7615
2	5.605	VB E	0.0500	107.83865	31.47351	4.0950
3	7.757	VB R	0.0687	135.45059	30.56368	5.1435

ESI-MS (*m/z*)

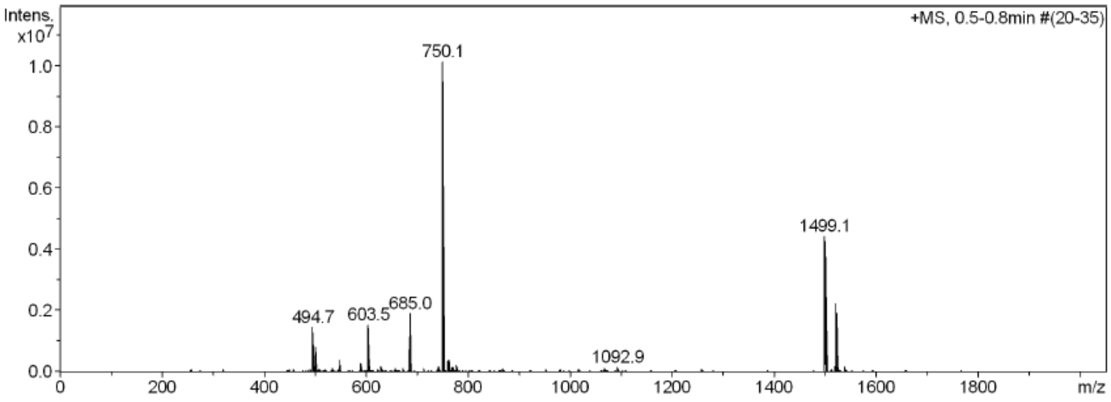

HRMS (m/z)

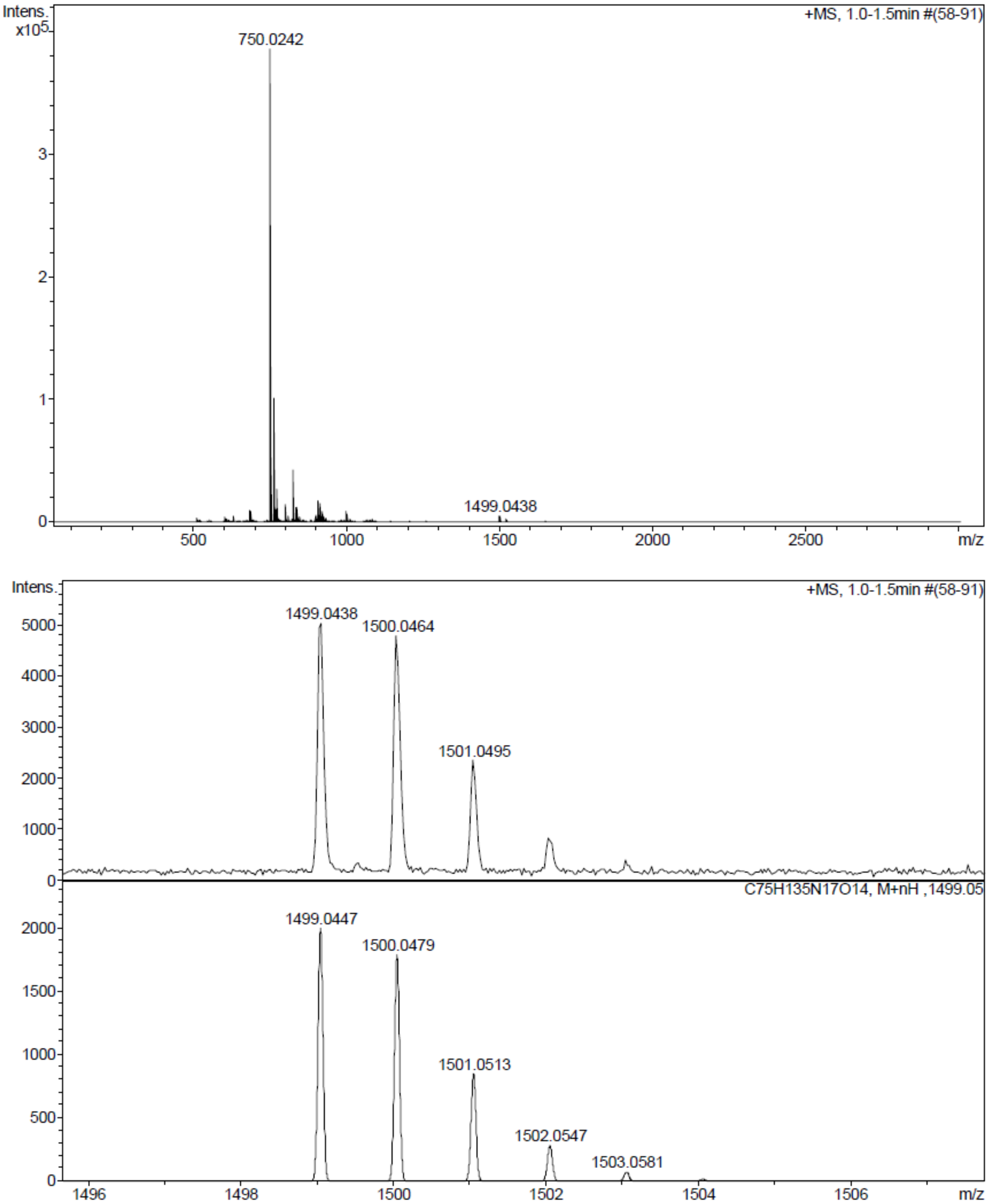
Intens.
x10^5
+MS, 1.0-1.5min #(58-91)
750.0242
1499.0438
m/z
Intens.
+MS, 1.0-1.5min #(58-91)
1499.0438
1500.0464
1501.0495
C75H135N17O14, M+nH ,1499.05
1499.0447
1500.0479
1501.0513
1502.0547
1503.0581
m/z

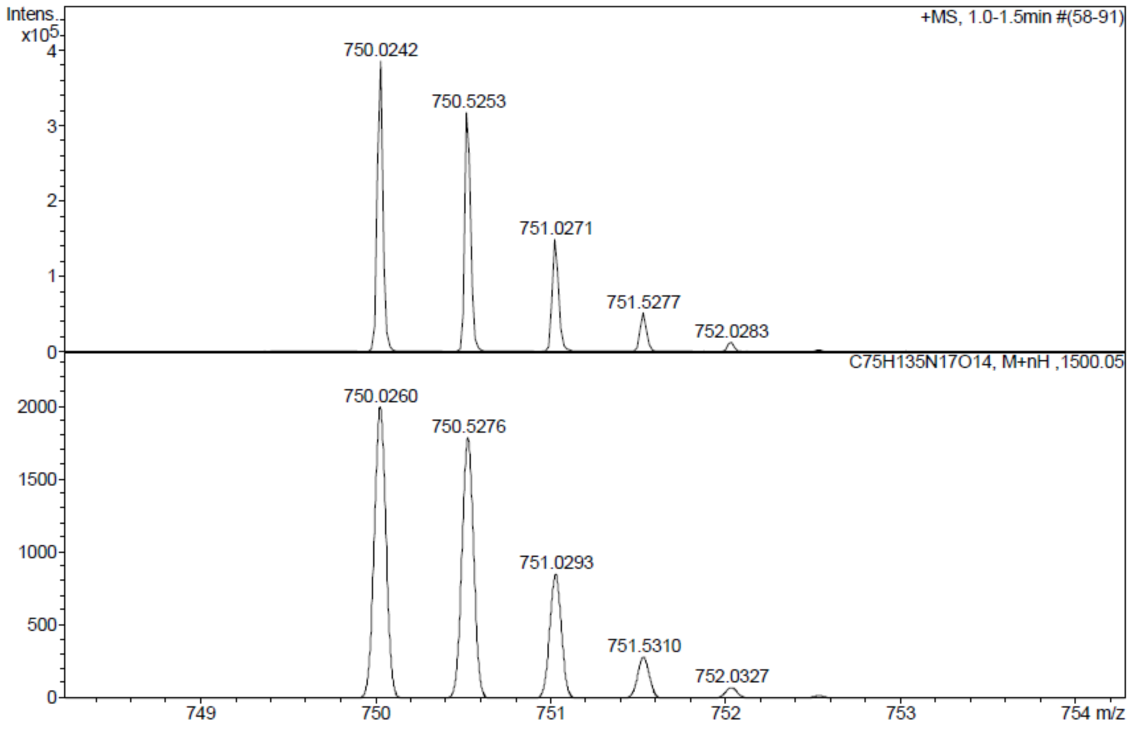

Intens.
x10^5
+MS, 1.0-1.5min #(58-91)
750.0242
750.5253
751.0271
751.5277
752.0283
C75H135N17O14, M+nH ,1500.05
750.0260
750.5276
751.0293
751.5310
752.0327
749
750
751
752
753
754 m/z

Ac-Lys-Lys-Leu-D-Phe-Lys-Lys-Lys(COC_3H_7)-Leu-Lys-Tyr-Leu-NH_2 (BP548)

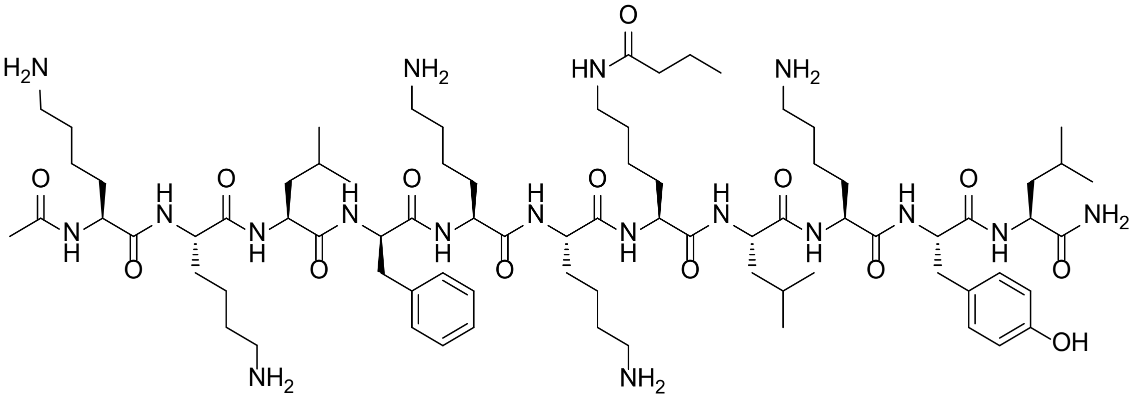

HPLC of purified peptide (λ=220 nm)

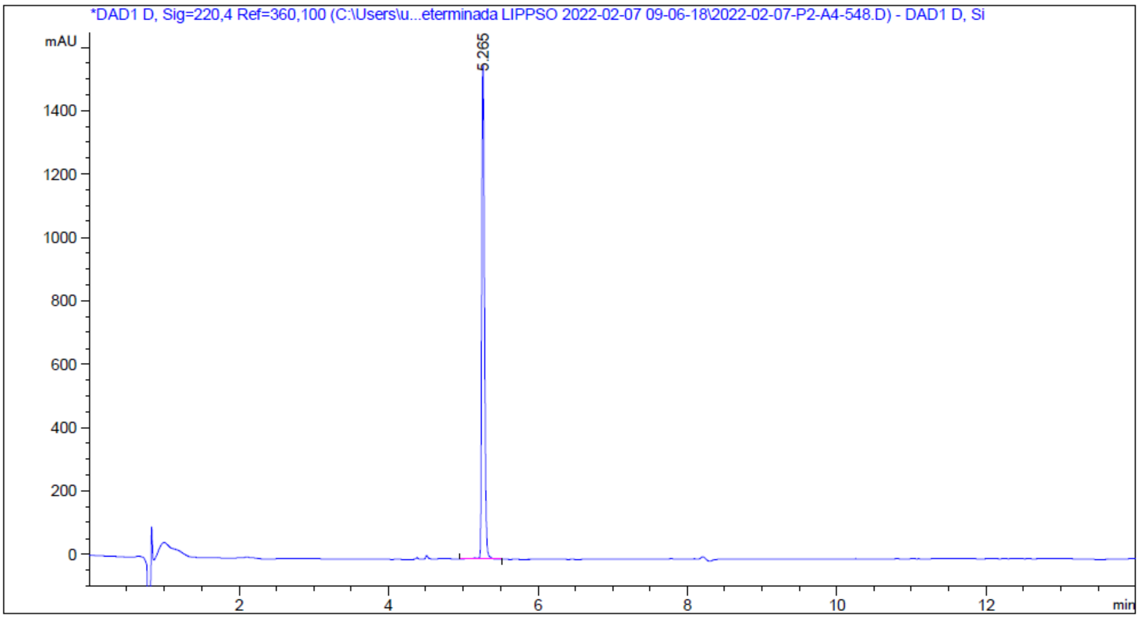

Peak #	RetTime [min]	Type	Width [min]	Area [mAU*s]	Height [mAU]	Area %
1	5.265	VB R	0.0390	3891.64526	1567.17554	100.0000

ESI-MS (*m/z*)

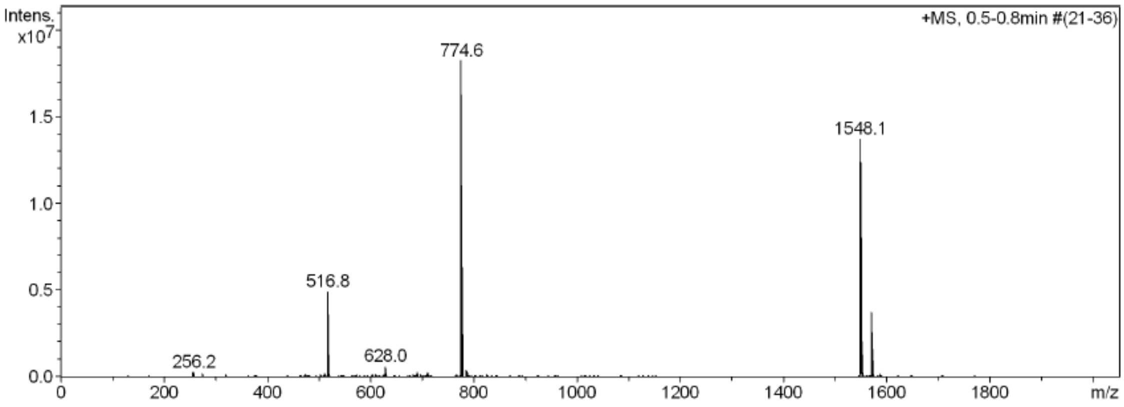

HRMS (*m/z*)

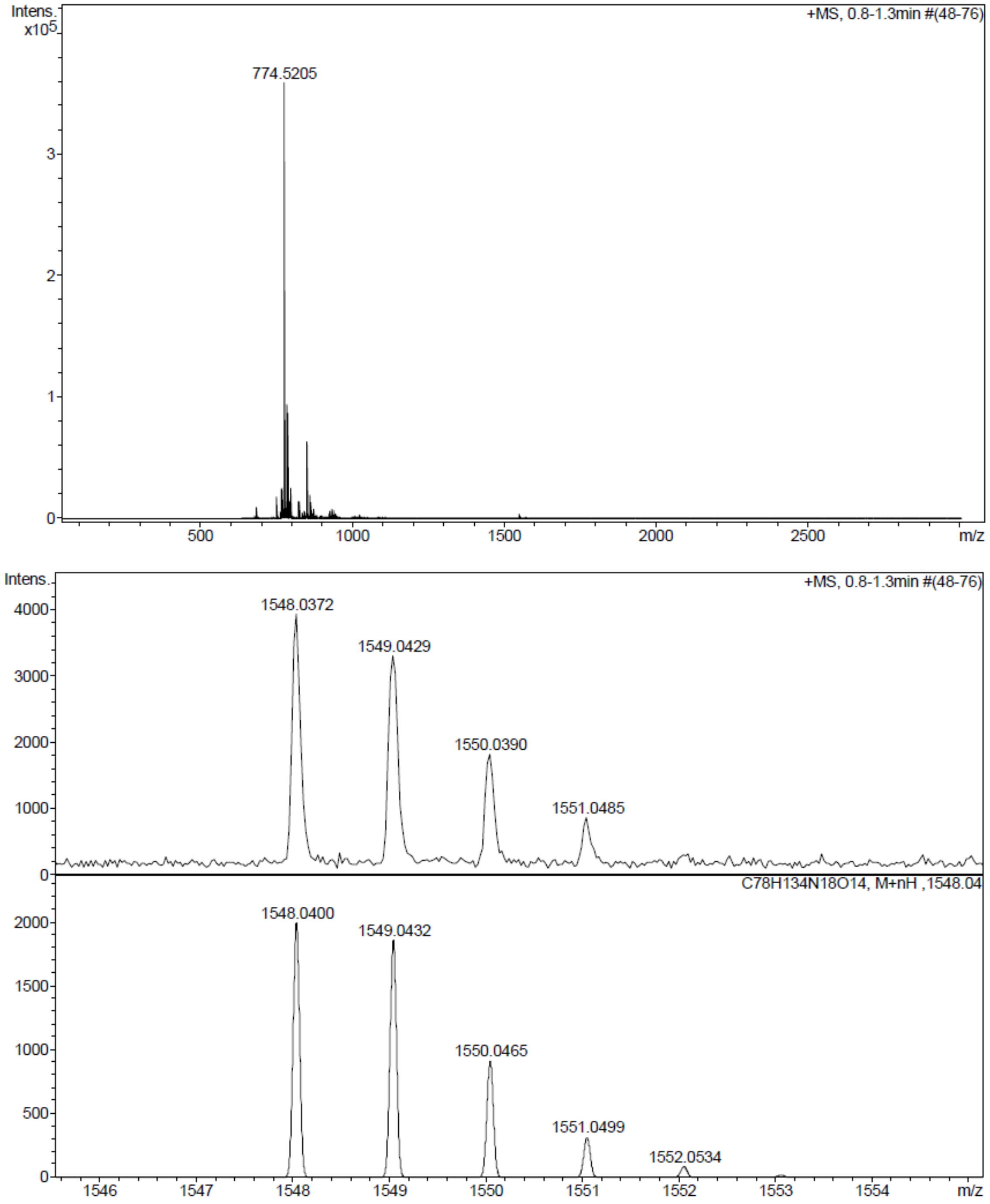
Intens.
x10^5
+MS, 0.8-1.3min #(48-76)
774.5205
m/z
Intens.
+MS, 0.8-1.3min #(48-76)
1548.0372
1549.0429
1550.0390
1551.0485
C78H134N18O14, M+nH ,1548.04
1548.0400
1549.0432
1550.0465
1551.0499
1552.0534
m/z

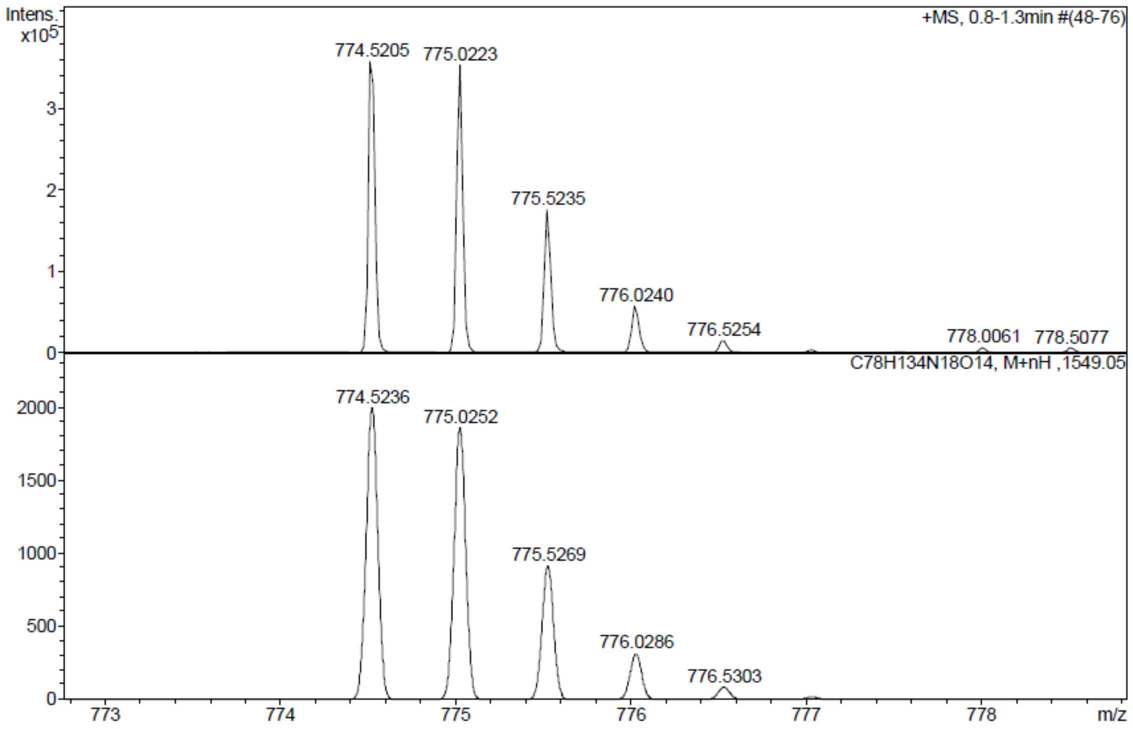
Intens.
x10^5
+MS, 0.8-1.3min #(48-76)
774.5205
775.0223
775.5235
776.0240
776.5254
778.0061
778.5077
C78H134N18O14, M+nH ,1549.05
774.5236
775.0252
775.5269
776.0286
776.5303
m/z

Ac-Lys-Lys-Lys(COC_3H_7)-D-Phe-Lys-Lys-Ile-Leu-Lys-Tyr-Leu-NH_2 (BP549)

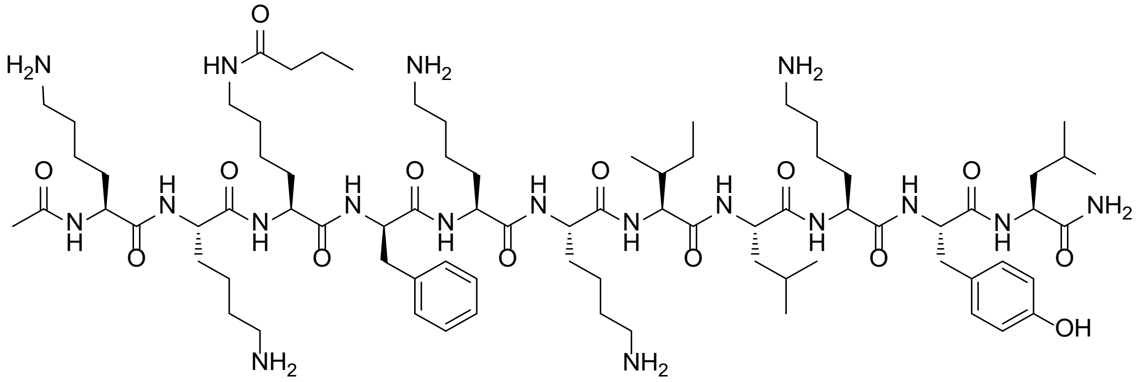

HPLC of purified peptide (λ=220 nm)

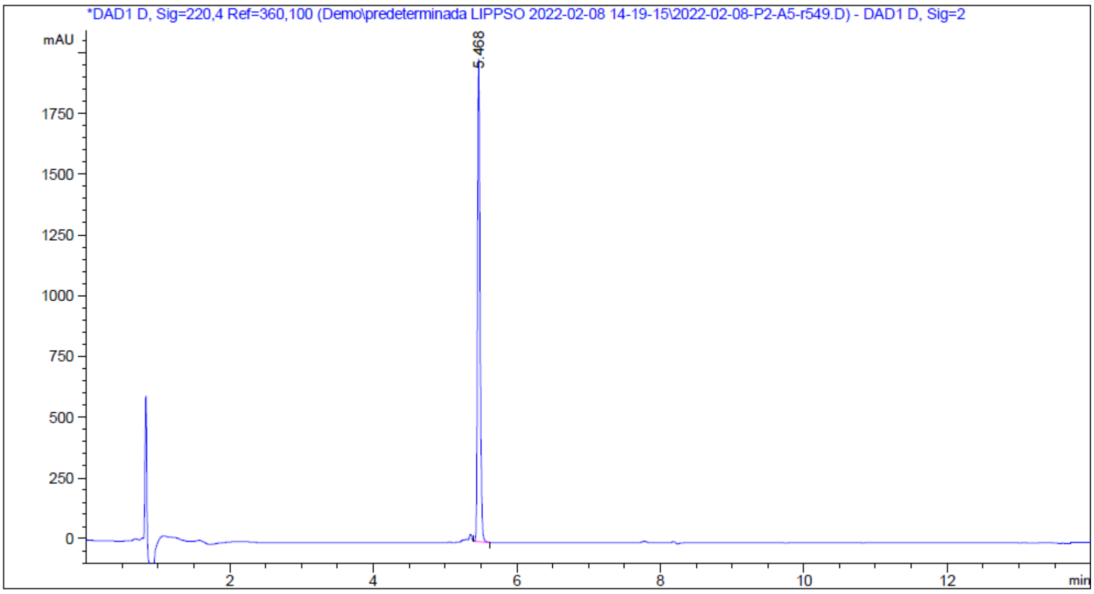

Peak #	RetTime [min]	Type	Width [min]	Area [mAU*s]	Height [mAU]	Area %
1	5.468	BB	0.0395	5033.11133	1991.42004	100.0000

ESI-MS (*m/z*)

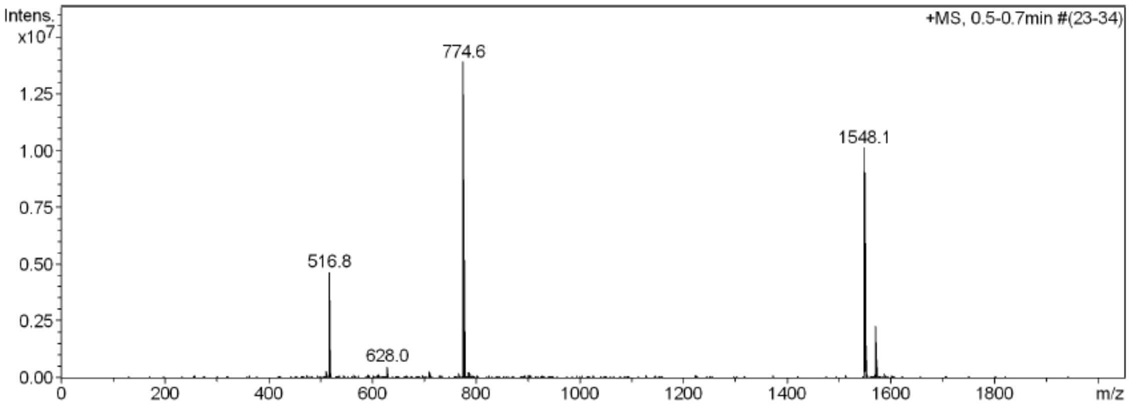

HRMS (*m/z*)

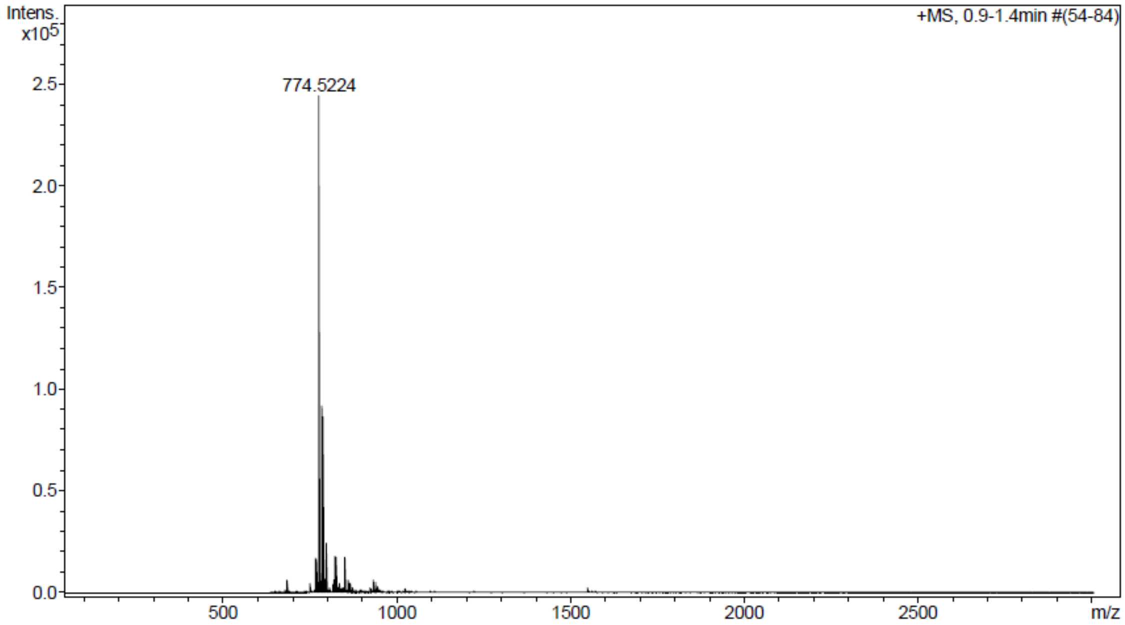

Intens.
x10^5
+MS, 0.9-1.4min #(54-84)
774.5224
2.5
2.0
1.5
1.0
0.5
0.0
500
1000
1500
2000
2500
m/z

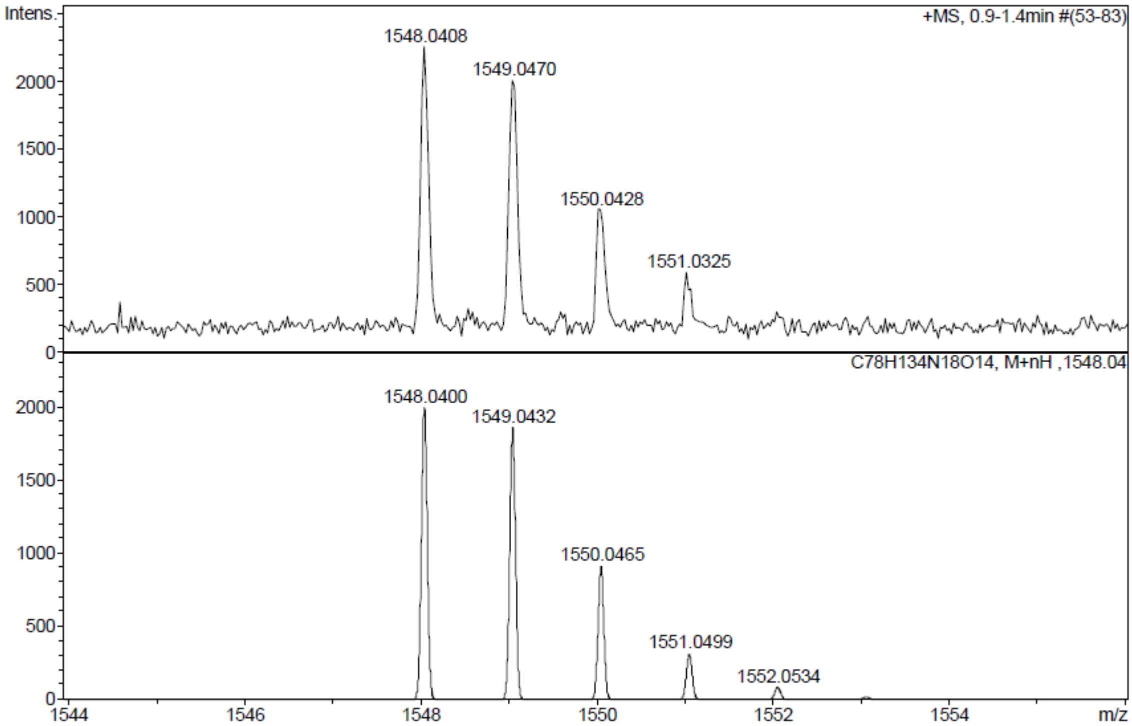

Intens.
+MS, 0.9-1.4min #(53-83)
1548.0408
1549.0470
1550.0428
1551.0325
C78H134N18O14, M+nH ,1548.04
1548.0400
1549.0432
1550.0465
1551.0499
1552.0534
2000
1500
1000
500
0
1544
1546
1548
1550
1552
1554
m/z

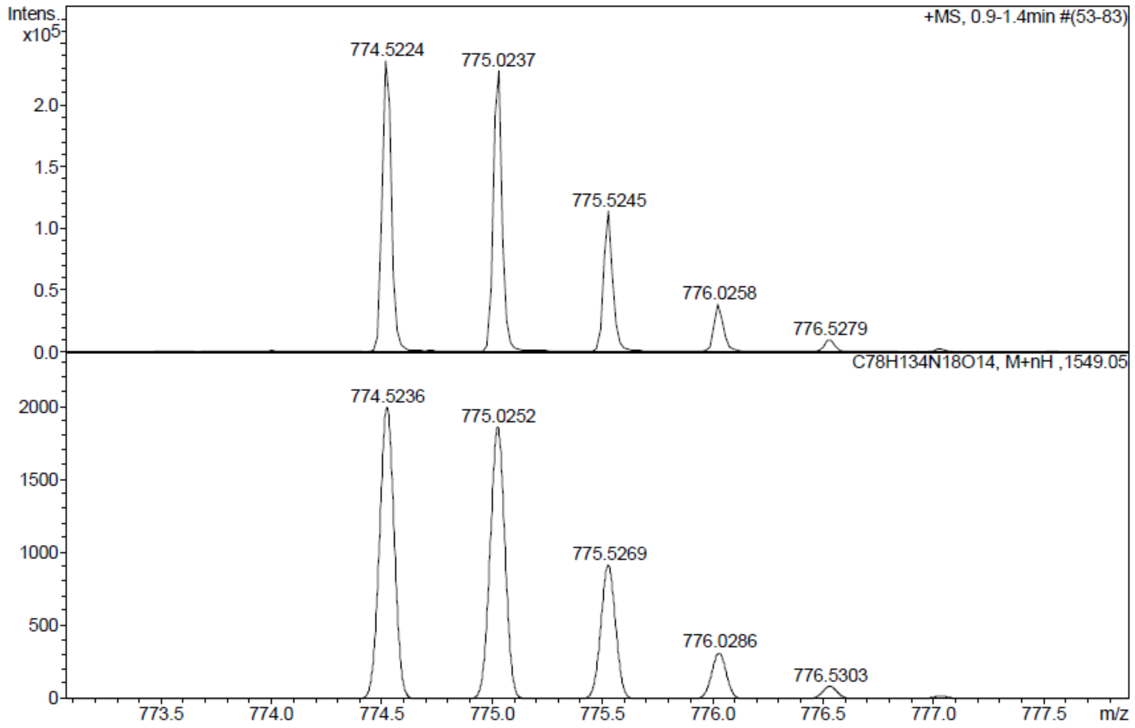
Intens.
x10^5
+MS, 0.9-1.4min #(53-83)
774.5224
775.0237
775.5245
776.0258
776.5279
C78H134N18O14, M+nH ,1549.05
774.5236
775.0252
775.5269
776.0286
776.5303
m/z

Ac-Lys-Lys-Leu-D-Lys(COC_3H_7)-Lys-Lys-Ile-Leu-Lys-Tyr-Leu-NH_2 (BP550)

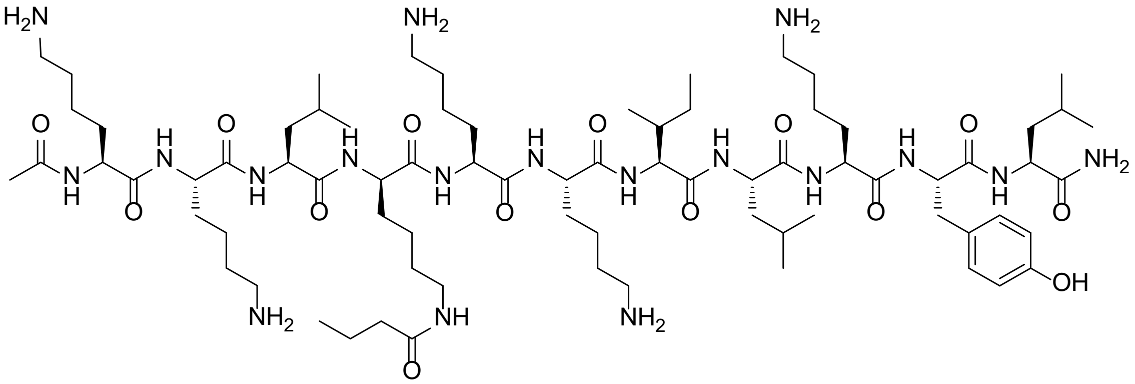

HPLC of purified peptide (λ=220 nm)

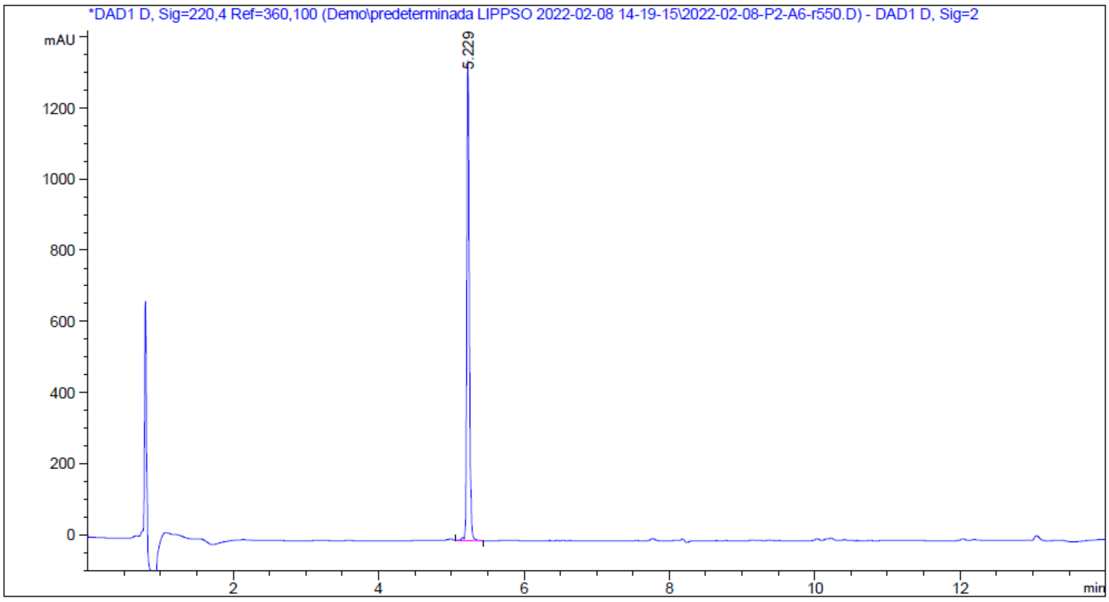

Peak #	RetTime [min]	Type	Width [min]	Area [mAU*s]	Height [mAU]	Area %
1	5.229	VB R	0.0379	3339.84082	1349.87939	100.0000

ESI-MS (*m/z*)

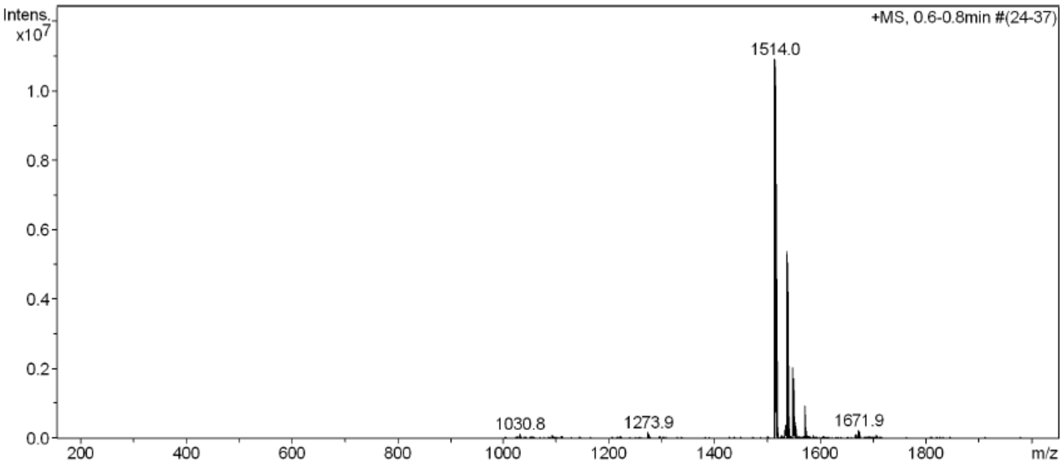

HRMS (*m/z*)

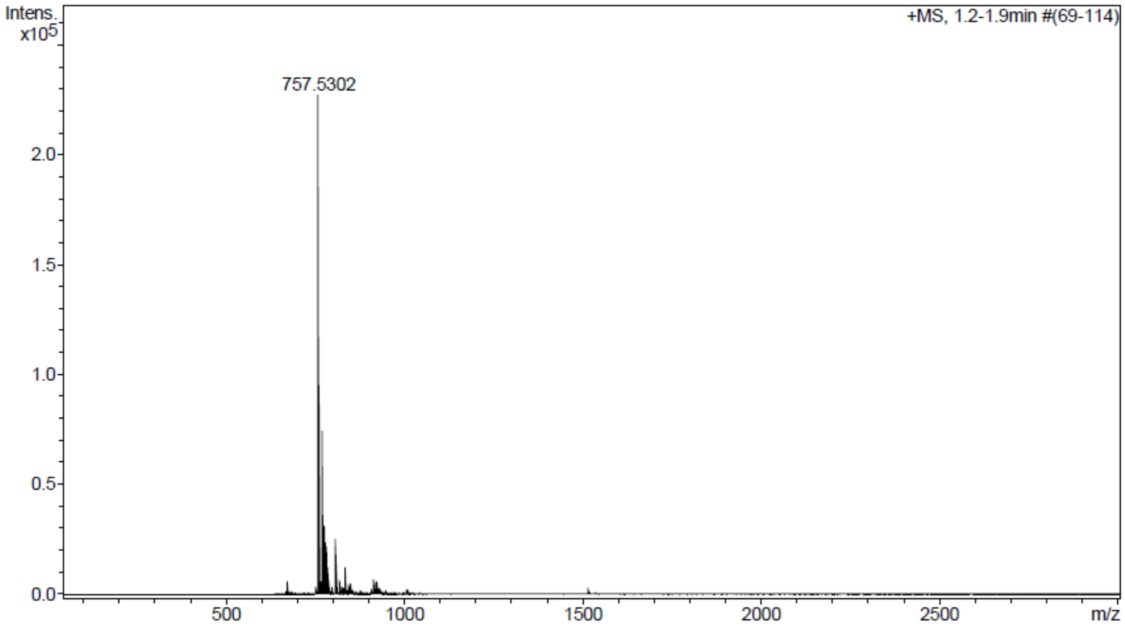

Intens.
x10^5
+MS, 1.2-1.9min #(69-114)
757.5302
2.0
1.5
1.0
0.5
0.0
500
1000
1500
2000
2500
m/z

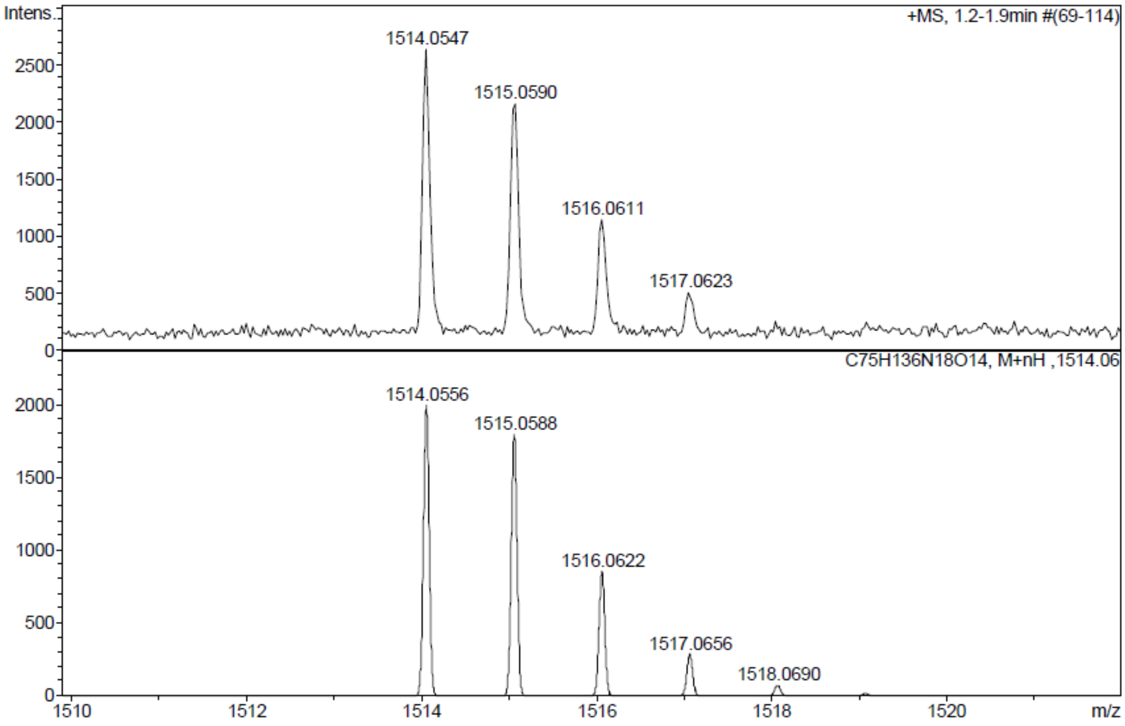

Intens.
+MS, 1.2-1.9min #(69-114)
1514.0547
1515.0590
1516.0611
1517.0623
2500
2000
1500
1000
500
0
C75H136N18O14, M+nH ,1514.06
1514.0556
1515.0588
1516.0622
1517.0656
1518.0690
2000
1500
1000
500
0
1510
1512
1514
1516
1518
1520
m/z

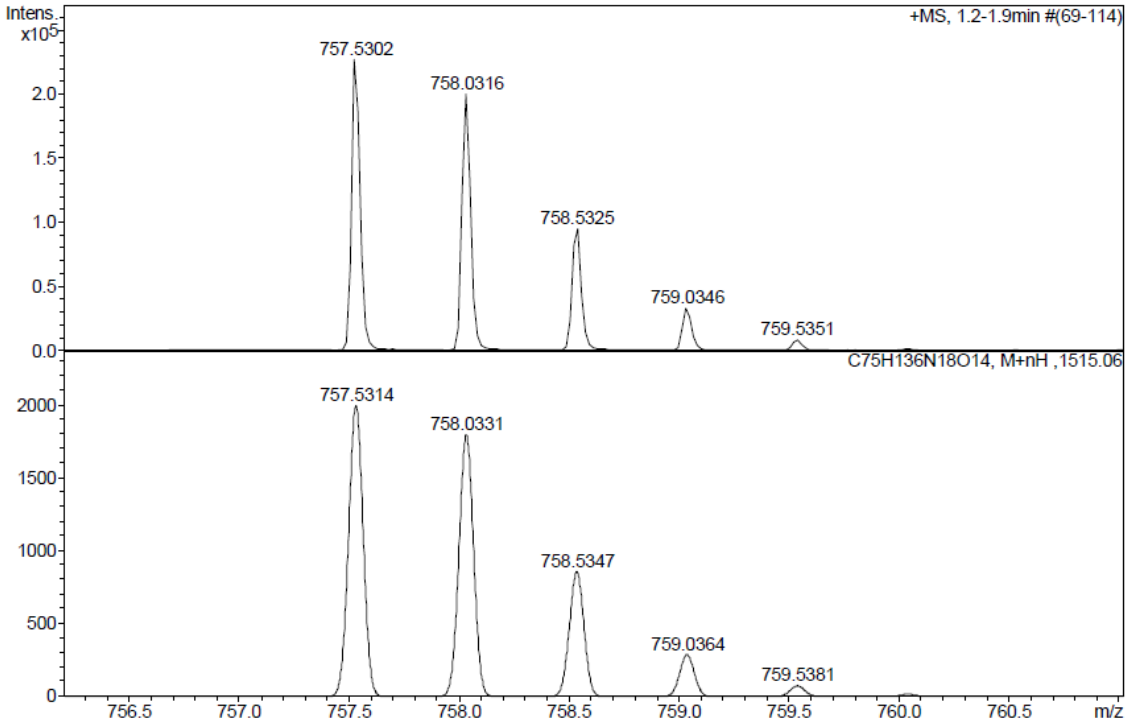

Intens.
x10^5
+MS, 1.2-1.9min #(69-114)
757.5302
758.0316
758.5325
759.0346
759.5351
2.0
1.5
1.0
0.5
0.0
C75H136N18O14, M+nH ,1515.06
757.5314
758.0331
758.5347
759.0364
759.5381
2000
1500
1000
500
0
756.5
757.0
757.5
758.0
758.5
759.0
759.5
760.0
760.5
m/z

CF-Lys-Lys-Leu-D-Phe-Lys-Lys(COC_3H_7)-Ile-Leu-Lys-Tyr-Leu-NH_2 (BP473-CF)

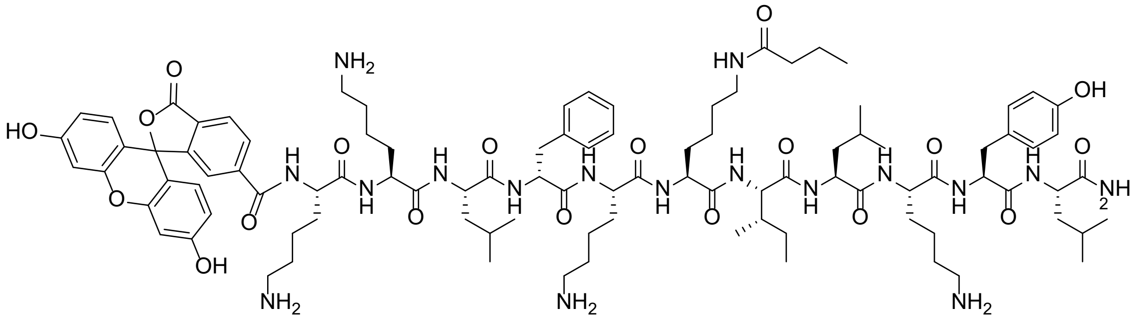

HPLC of purified peptide (λ=220 nm)

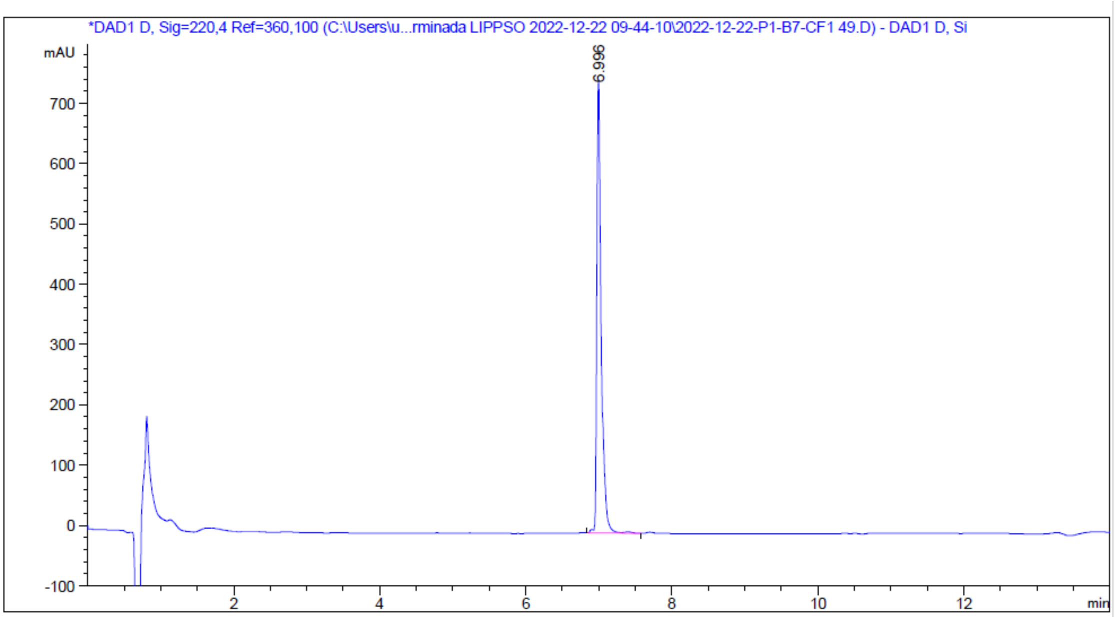

Peak #	RetTime [min]	Type	Width [min]	Area [mAU*s]	Height [mAU]	Area %
1	6.996	VV R	0.0598	3086.48730	753.79718	100.0000

ESI-MS (*m/z*)

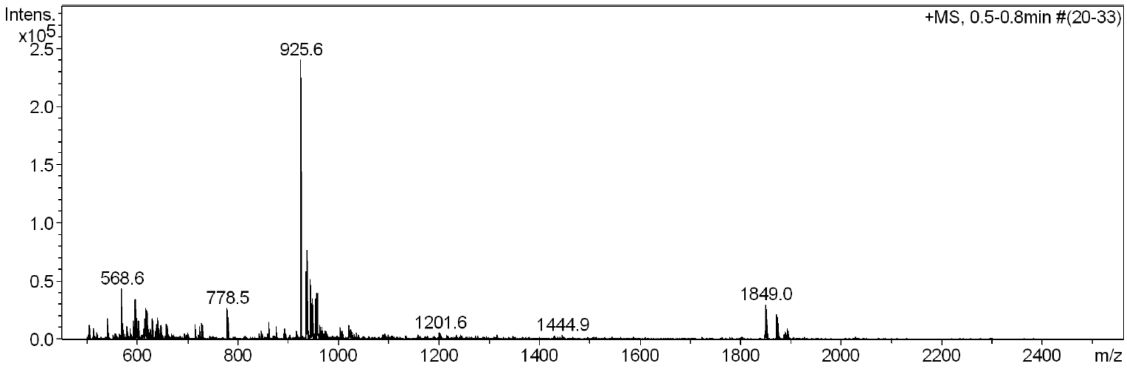

HRMS (*m/z*)

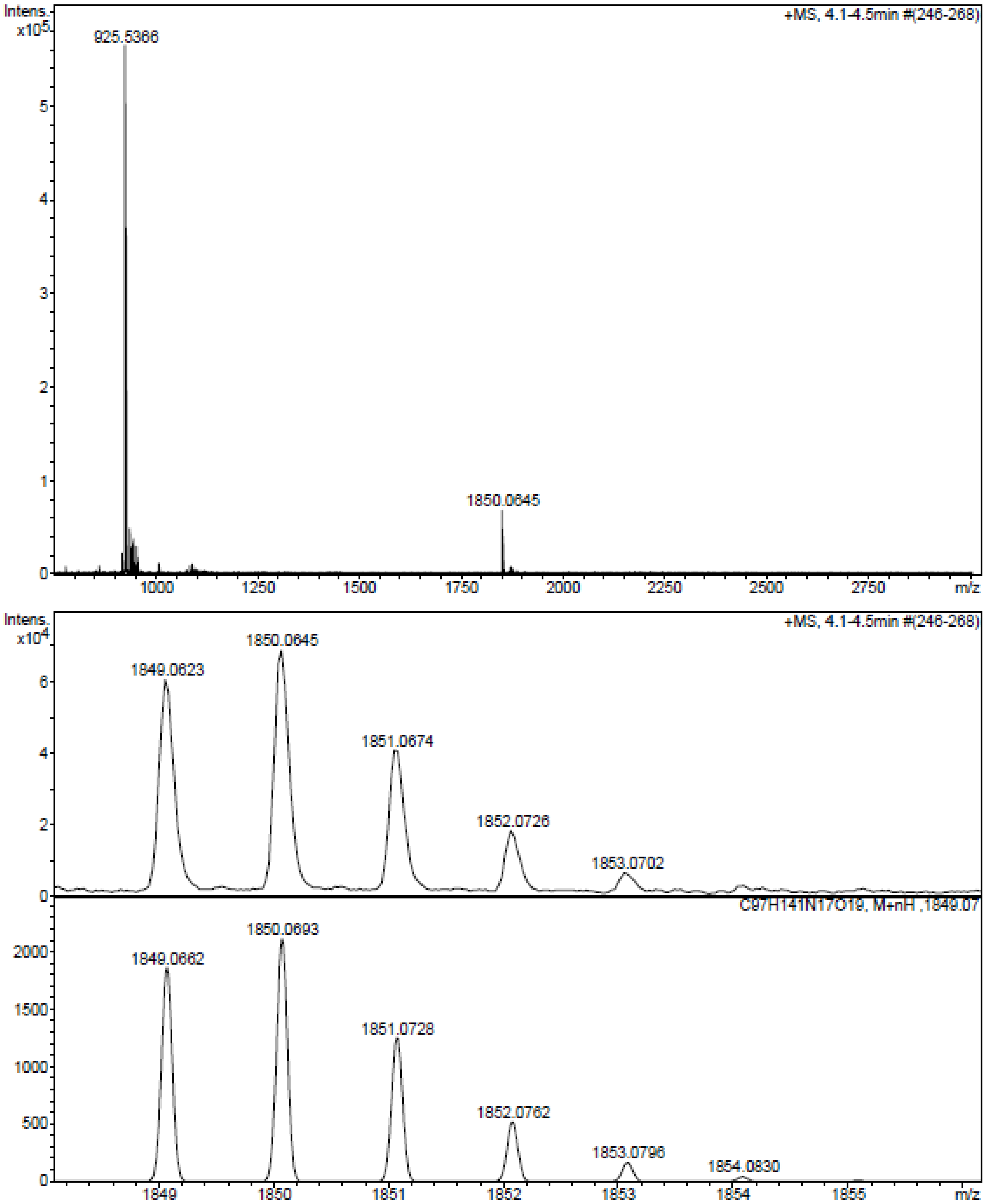
Intens. x10^5
925.5366
1850.0645
+MS, 4.1-4.5min #(246-268)
m/z
Intens. x10^4
1849.0623
1850.0645
1851.0674
1852.0726
1853.0702
+MS, 4.1-4.5min #(246-268)
C97H141N17O19, M+nH ,1849.07
1849.0662
1850.0693
1851.0728
1852.0762
1853.0796
1854.0830
m/z

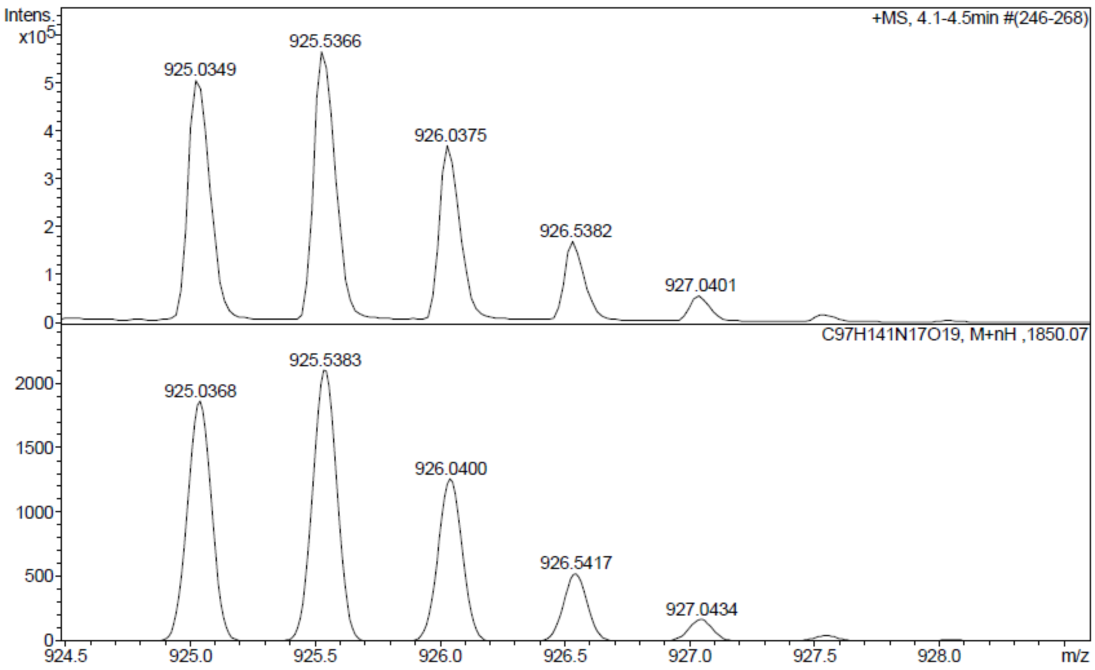
Intens.
x10^5
+MS, 4.1-4.5min #(246-268)
925.0349
925.5366
926.0375
926.5382
927.0401
C97H141N17O19, M+nH ,1850.07
925.0368
925.5383
926.0400
926.5417
927.0434
m/z

Ac-Lys(CF)-Lys-Lys-Leu-D-Phe-Lys-Lys(COC_3H_7)-Ile-Leu-Lys-Tyr-Leu-NH_2 (BP473-K(CF))

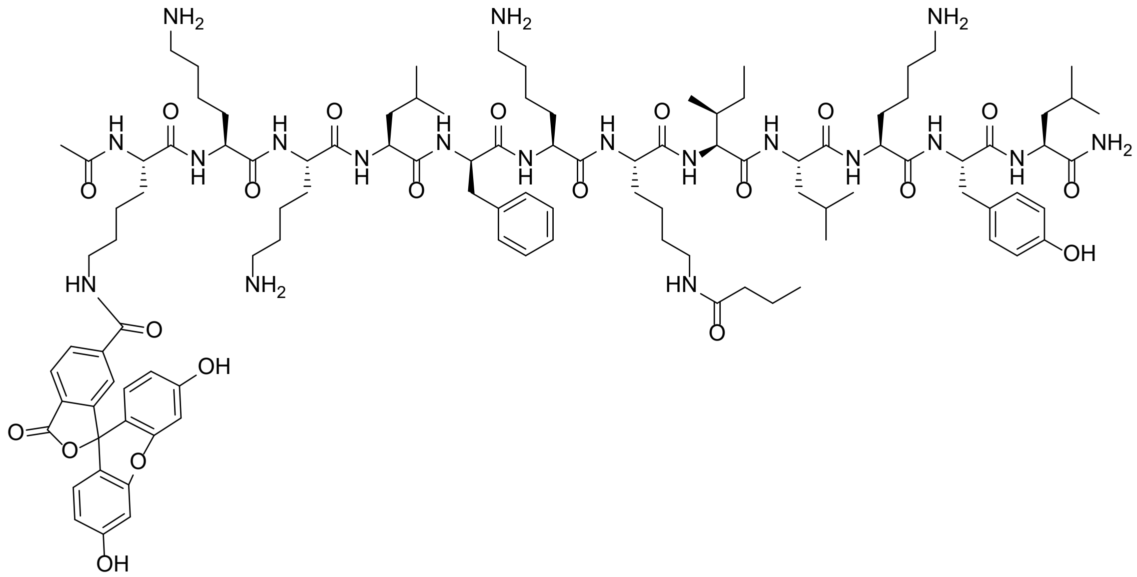

HPLC of purified peptide (λ=220 nm)

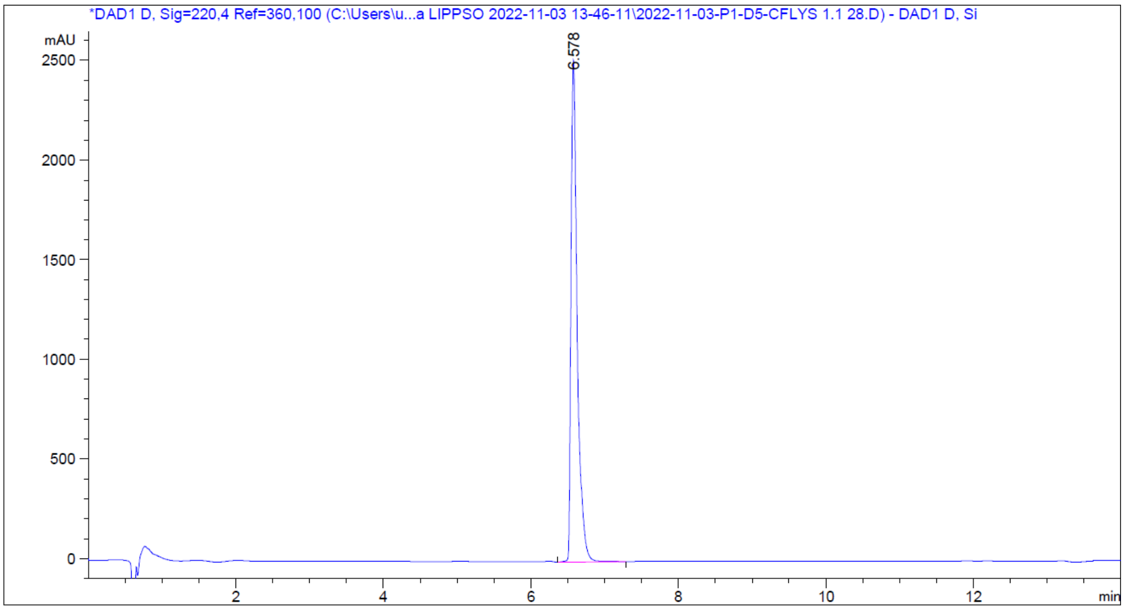

Peak #	RetTime [min]	Type	Width [min]	Area [mAU*s]	Height [mAU]	Area %
1	6.578	BB	0.0904	1.47772e4	2520.29614	100.0000

ESI-MS (*m/z*)

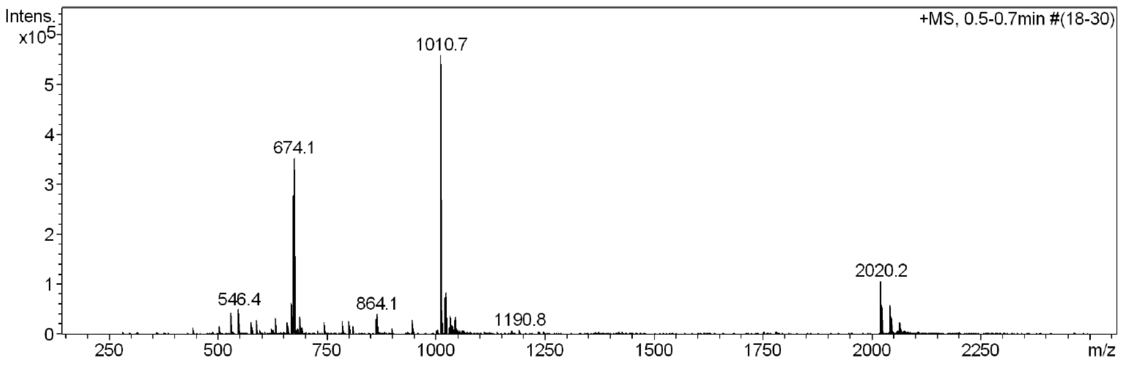

HRMS (*m/z*)

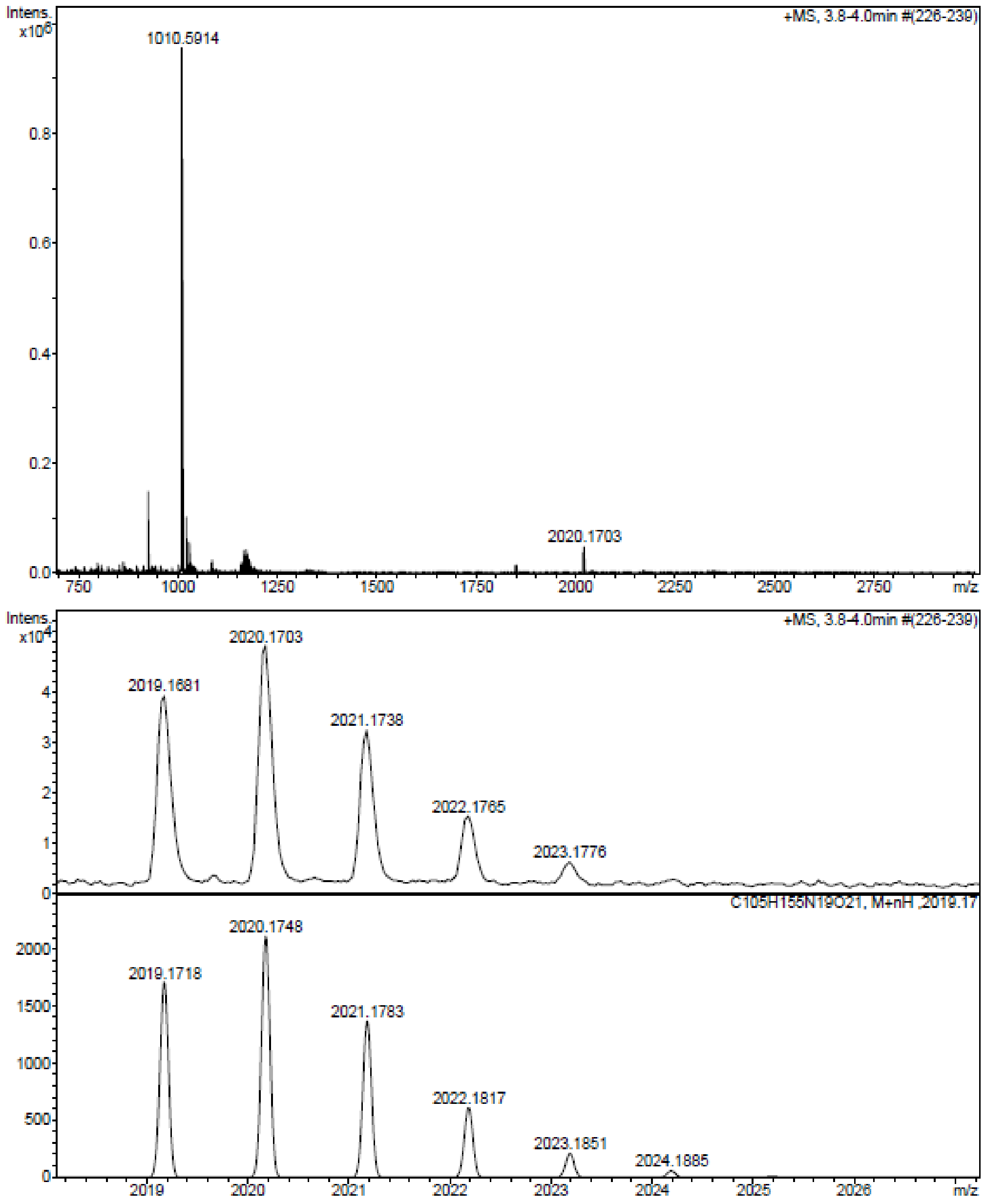

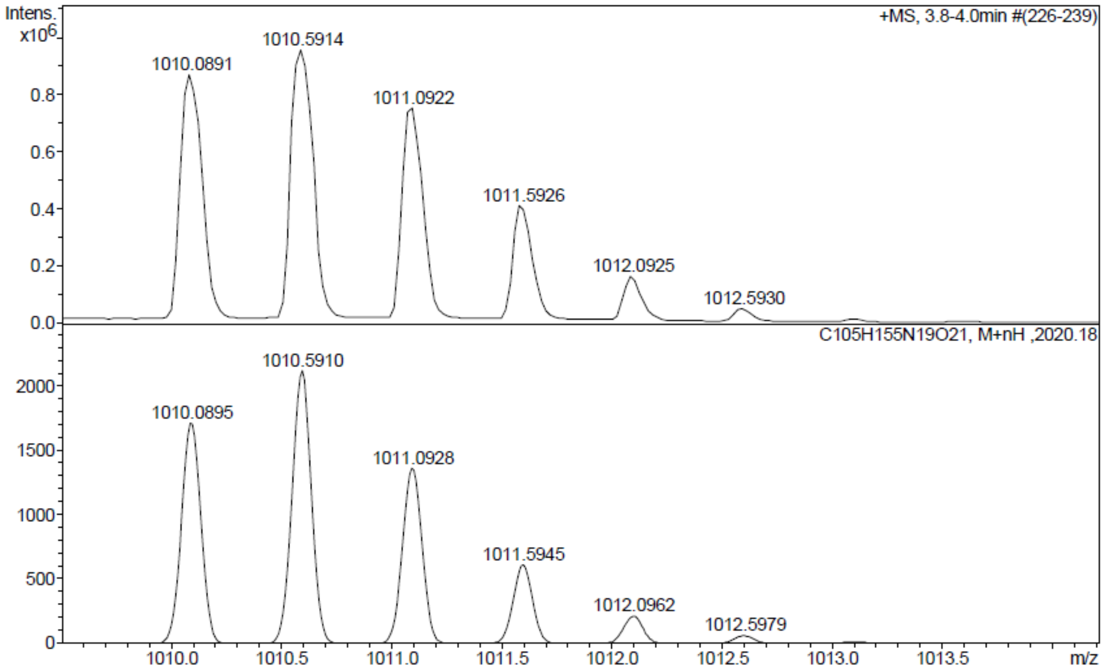
Intens.
x10^6
+MS, 3.8-4.0min #(226-239)
1010.0891
1010.5914
1011.0922
1011.5926
1012.0925
1012.5930
C105H155N19O21, M+nH ,2020.18
1010.5910
1010.0895
1011.0928
1011.5945
1012.0962
1012.5979
m/z
